# Learning of Artificial Sensation Through Long-Term Home Use of a Sensory-Enabled Prosthesis

**DOI:** 10.3389/fnins.2019.00853

**Published:** 2019-08-21

**Authors:** Ivana Cuberovic, Anisha Gill, Linda J. Resnik, Dustin J. Tyler, Emily L. Graczyk

**Affiliations:** ^1^Department of Biomedical Engineering, Case Western Reserve University, Cleveland, OH, United States; ^2^Louis Stokes Cleveland VA Medical Center, Cleveland, OH, United States; ^3^Providence VA Medical Center, Providence, RI, United States; ^4^Department of Health Services, Policy, and Practice, Brown University, Providence, RI, United States

**Keywords:** neural prosthesis, touch perception, proprioception, learning, embodiment/bodily experience, amputation – rehabilitation, home use, phantom limb experience

## Abstract

Upper limb prostheses are specialized tools, and skilled operation is learned by amputees over time. Recently, neural prostheses using implanted peripheral nerve interfaces have enabled advances in artificial somatosensory feedback that can improve prosthesis outcomes. However, the effect of sensory learning on artificial somatosensation has not been studied, despite its known influence on intact somatosensation and analogous neuroprostheses. Sensory learning involves changes in the perception and interpretation of sensory feedback and may further influence functional and psychosocial outcomes. In this mixed methods case study, we examined how passive learning over 115 days of home use of a neural-connected, sensory-enabled prosthetic hand influenced perception of artificial sensory feedback in a participant with transradial amputation. We examined perceptual changes both within individual days of use and across the duration of the study. At both time scales, the reported percept locations became significantly more aligned with prosthesis sensor locations, and the phantom limb became significantly more extended toward the prosthesis position. Similarly, the participant’s ratings of intensity, naturalness, and contact touch significantly increased, while his ratings of vibration and movement significantly decreased across-days for tactile channels. These sensory changes likely resulted from engagement of cortical plasticity mechanisms as the participant learned to use the artificial sensory feedback. We also assessed psychosocial and functional outcomes through surveys and interviews, and found that self-efficacy, perceived function, prosthesis embodiment, social touch, body image, and prosthesis efficiency improved significantly. These outcomes typically improved within the first month of home use, demonstrating rapid benefits of artificial sensation. Participant interviews indicated that the naturalness of the experience and engagement with the prosthesis increased throughout the study, suggesting that artificial somatosensation may decrease prosthesis abandonment. Our data showed that prosthesis embodiment was intricately related to naturalness and phantom limb perception, and that learning the artificial sensation may have modified the body schema. As another indicator of successfully learning to use artificial sensation, the participant reported the emergence of stereognosis later in the study. This study provides the first evidence that artificial somatosensation can undergo similar learning processes as intact sensation and highlights the importance of sensory restoration in prostheses.

## Introduction

Tool use is a ubiquitous human trait. Prostheses for upper limb amputees are considered to be a special case of tool use, because their purpose is to replace a missing body part rather than to augment normal human capabilities. A classically studied goal of upper limb prosthesis rehabilitation has been recovery of grasping and dexterous manipulation. To that end, considerable efforts have gone into the development of mechanically dexterous prosthetic limbs (Weir and Sensinger, 2009; Belter et al., 2013; Bajaj et al., 2018) and intuitive algorithms to control them (Scheme and Englehart, 2011; Dalley et al., 2012; Hargrove et al., 2017; Segil et al., 2017), with some of these technologies becoming commercially available. More recently, somatosensation has also been restored in upper limb prostheses, either through non-invasive electrocutaneous and vibrotactile techniques (Dietrich et al., 2012, 2018; Clemente et al., 2016) or through implanted neural interfaces (Raspopovic et al., 2014; Tan et al., 2014; Davis et al., 2016). Multiple groups have investigated direct electrical stimulation of the remaining nerves as a means of restoring sensation of the missing hand to upper limb amputees (Raspopovic et al., 2014; Tan et al., 2014; Davis et al., 2016). In addition to quantifying the evoked percepts (Tan et al., 2014; Graczyk et al., 2016, 2018a), these groups have also shown marked improvements in performance of functional tasks, such as object identification (Schiefer et al., 2016), object feature discrimination (Horch et al., 2011; Raspopovic et al., 2014; Oddo et al., 2016; Schiefer et al., 2018), and closed-loop control (Wendelken et al., 2017; Valle et al., 2018), when sensory feedback is provided.

Like all tool use, skilled prosthesis use develops over time and often requires training. The relationship between training and general skill acquisition has been quantified through the learning curve (Newell and Rosenbloom, 1981; DeKeyser, 2015). In its simplest form, the learning curve shows that markers of skill, such as increases in accuracy, decreases in errors, and decreases in cognitive effort, improve with training over extended durations of time (Newell and Rosenbloom, 1981; DeKeyser, 2015). Active learning occurs as the task or skill is explicitly practiced through a training regimen. In contrast, passive learning occurs as the task or skill is performed as needed during daily activities.

Learning skilled tool use involves neural changes in both the motor and sensory systems. Perceptual learning is enabled by plasticity in the sensory cortex (Gilbert et al., 2001) and involves increases in the size of the representation of a stimulus in the sensory cortex, narrowing of the selectivity of tuned cells, changes in the temporal relationships of neuronal responses, and shifts in processing from higher to lower sensory cortices (Gilbert et al., 2001; Hoffman and Logothetis, 2009). Indeed, numerous studies have shown expansion of somatosensory cortical regions to enable trained sensorimotor skills, such as reading Braille (Pascual-Leone and Torres, 1993), playing instruments (Elbert et al., 1995), or understanding speech with a cochlear prosthesis (Fallon et al., 2008).

Tool embodiment is also intricately related to sensory learning, as they both involve similar neural mechanisms. Multiple groups have shown that both referred sensations on the residual limb and localized percepts on the missing limb can increase embodiment of a prosthesis (Ehrsson et al., 2008; Dietrich et al., 2012; D’Alonzo et al., 2015; Graczyk et al., 2018b, 2019; Marasco et al., 2018; Page et al., 2018; Valle et al., 2018). Prosthesis embodiment is an important psychosocial outcome that is related to positive prosthesis outcomes (Murray, 2004; Graczyk et al., 2019). It is defined as both the conscious perception of tool inclusion within one’s bodily borders and the preconscious sensorimotor processing of the tool as if it belonged to the body (Gallagher, 2005; Haggard and Wolpert, 2005; Arzy et al., 2006; Giummarra et al., 2008; Longo et al., 2008; de Vignemont, 2010b, 2011). Although the criteria necessary for embodiment to occur vary through the literature, embodiment is generally understood to emerge from multisensory integration among spatiotemporally coincident stimuli (de Vignemont, 2010b). Embodiment alters the processing of sensory events in both peri-personal and personal space (Iriki et al., 1996; Galli et al., 2015; Miller et al., 2017) and modifies the body schema, which is the preconscious, dynamic sensorimotor representation of the body (Cardinalli et al., 2009; Jovanov et al., 2015). Because they replace lost body parts, prostheses may be truly incorporated into the body schema, rather than operating on the level of bodily extension like most tools (de Preester and Tsakiris, 2009; de Vignemont, 2010b).

The role of learning in interpreting artificial sensory feedback has been studied most extensively in audition with the advent of cochlear prostheses (Watson, 1991; Fu et al., 2005; Fu and Galvin, 2007, 2012; Oba et al., 2011). With cochlear prostheses, learning is defined as improvements in auditory perception and interpretation over time, and differences between passive and active perceptual learning have been quantified (Fu and Galvin, 2012). Both passive and active learning regimens are associated with corresponding cortical plasticity (Tremblay et al., 1997, 1998; Fu and Galvin, 2007; Gentner and Margoliash, 2009; Sharma and Dorman, 2012). While auditory perception through cochlear prostheses can improve with passive learning, these improvements typically plateau in 3–12 months (Watson, 1991; Fu and Galvin, 2007, 2012). Additional improvements in perception require active training interventions, such as training in particular noise environments (Fu et al., 2005; Fu and Galvin, 2007; Oba et al., 2011) or training to recognize specific phonemes (Fu et al., 2005).

However, the effect of sensory learning on the perception and utilization of artificial somatosensory percepts evoked by electrical stimulation in upper limb prostheses has not been studied. For artificial somatosensation in prosthetic limbs, studies primarily investigate sensation in controlled laboratory environments, where laboratory visits are sporadic and only last for a few hours or days at a time. Given prior studies on passive learning in cochlear prostheses, which indicate that passive learning can continue shaping auditory perception for up to a year (Watson, 1991; Fu and Galvin, 2007, 2012), studies of sensory learning in upper limb prostheses may similarly require months to years of extended usage. To our knowledge, there has only been one study in which participants received artificial somatosensation from implanted nerve interfaces for multiple days in a row. This study involved independent usage of a prosthesis with stimulation-evoked sensory feedback in home and community settings for up to 2 weeks in two participants (Graczyk et al., 2018b). We found that sensory detection thresholds were stable over this duration, but did not quantify whether any aspects of the sensations changed over time with continued exposure to stimulation. Sensory learning may also influence functional and psychosocial outcomes. In our prior home use study, we also found that sensory feedback impacted the psychosocial experience of prosthesis embodiment, confidence, and perceived efficiency. While we found that function was better with sensation than without in our prior home study, it did not improve over the short duration of the study. Because the study was brief relative to the passive learning interval, we were not able to investigate the time course of these changes or whether they had plateaued.

Thus, the purpose of this study is to investigate whether artificial somatosensory feedback can be learned over time. We define learning as changes in the perception and utilization of artificial somatosensation and changes in user outcomes due to prolonged exposure to sensory stimulation. We examined the impact of extended daily usage of the sensory-enabled prosthesis on quantitative metrics of the perception of evoked somatosensation, perception of the phantom limb, psychosocial outcomes, and functional outcomes. We also conducted a qualitative analysis of data from in-person interviews. We hypothesized that extended usage of a sensory-enabled prosthesis would correspond with sensory percept changes to better align with information transduced by the prosthesis sensors. We further hypothesized that extended usage would yield better functional and psychosocial outcomes.

## Materials and Methods

### Subject

One adult male with unilateral transradial limb loss participated in this trial. He sustained a traumatic right transradial amputation approximately 3 inches distal to the elbow in a workplace accident in 2004 and was right hand dominant prior to amputation. The participant was implanted with 8-channel Flat Interface Nerve Electrodes (FINEs) around his median and radial nerves in 2013. He participated in a previous short-term home study in months 41–42 post-implant (Graczyk et al., 2018b). Data for the current home study was collected in months 71–75 post-implant. The participant did not report any instances of phantom pain during this time. All study devices and procedures were reviewed and approved by the U.S. Food and Drug Administration Investigational Device Exemption, the Cleveland Department of Veterans Affairs Medical Center Institutional Review Board, and the Department of the Navy Human Research Protection Program. All study procedures and experiments were performed in accordance with relevant guidelines and regulations of these institutions. Written informed consent was obtained from the subject.

### Home Use System

**FIGURE 1:**
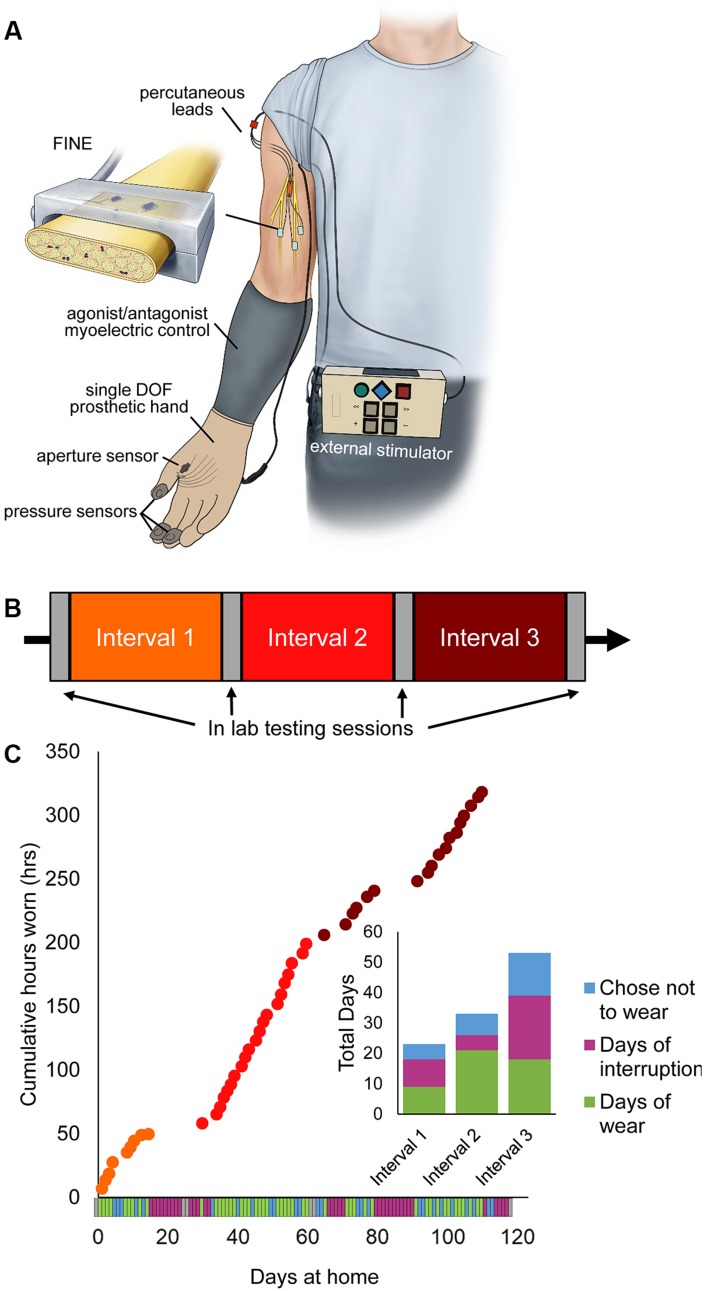
Home system for sensory restoration and usage throughout the study. **(A)** Home system for sensory restoration. Implanted peripheral nerve cuff electrodes delivered artificial somatosensory feedback of touch and proprioception corresponding to sensors on a hand prosthesis. This panel is reproduced from Graczyk et al. (2018b) with minor revisions (CC BY 4.0, https://creativecommons.org/licenses/by/4.0/). **(B)** Study design. The three at-home study intervals were flanked by in-lab testing sessions. **(C)** Usage of the restored sensation varied throughout the study (*n* = 115 days in total). Days of wear were interspersed with days of non-wear, either due to interruptions or choice. Interruptions included breakage of system components that necessitated repair by the study team, illnesses, and urgent personal matters (Supplementary Table S1).

The home use system included a portable neurostimulator and associated hardware for providing artificial somatosensory feedback with a single degree-of-freedom (DOF) prosthesis (Figure 1A). The subject wore his own, clinically fit prosthetic socket and used his standard settings for agonist/antagonist myoelectric control. An OttoBock VariPlus Speed prosthetic hand was augmented by embedding force-sensitive resistors in the tips of the thumb, index, and middle fingers using medical grade silicone adhesive. An aperture sensor was mounted in the prosthetic hand to measure the opening span of the hand’s single DOF. The installed sensors sent analog signals via a cable to the neurostimulator, which was worn about the waist in a small pack. The neurostimulator translated the incoming pressure and aperture data into stimulation pulse trains and sent the stimulation trains to the subject’s implanted FINEs via a cable to his percutaneous leads. Stimulation pulses were cathode-first, biphasic, and charge-balanced. Each sensor in the prosthetic hand corresponded to a single electrode contact in the FINEs. A set of three electrode contacts inside the FINE were used as the return current path for all active contacts. Increases in pressure or decreases in hand aperture were linearly scaled to stimulation pulse frequency. Full details of the home use system are presented in an earlier publication (Graczyk et al., 2018b).

### Study Design

The study used a quasi-experimental time series design with three periods of home use, each separated by 2 days of in-lab testing (Figure 1B). The participant also completed in-lab testing at the start and end of the study. The total duration of the study was 115 days, including in-lab testing. Each period of home use was intended to be 1-month in duration. However, due to interruptions throughout the study, the durations of home use periods were modified (Figure 1C). Interruptions included system component breakages, illnesses, and other personal emergencies (Supplementary Table S1). The participant wore the system a home for 9, 21, and 19 days in intervals 1, 2, and 3, respectively. The average wear time of the system, based on onboard system logs, was 6.7 ± 0.25 h/day (mean ± SEM). There was no difference in wear time based on study interval (1-way ANOVA, *p* = 0.065).

### Outcome Measures

Onboard usage logs, surveys, and functional outcomes were obtained at various time courses throughout the study (Table 1). Two general categories of metrics, at-home and in-lab, were collected. At-home metrics included surveys administered daily or weekly on days the participant wore the system and onboard usage logs, which recorded timestamps for system on and off periods and continuously recorded prosthesis sensor activity. In-lab measures consisted of surveys and functional tests administered during the participant’s laboratory visits. The metrics utilized in this study were a subset of the metrics obtained during our prior home study, and details of each metric can be found in the previous publication (Graczyk et al., 2018b).

**TABLE 1:** Administered measures and time courses of data collection.

	**Metric**	**Measure of**	**Administered**			
			**Daily**		**Weekly**	**Monthly**
			**AM**	**PM**	**PM**	
Sensation	Sensation location survey	• Sensation location	✓	✓		
	
	Sensation quality survey	• Sensation quality	✓	✓		
	
	Perceived limb length	• Phantom position	✓	✓		

Psychosocial	PEM	• Embodiment • Perception of abilities • Social interactions • Body image • Prosthesis efficiency				✓
	
	PEM (short form)	• Embodiment • Perception of abilities • Social interactions • Prosthesis efficiency		✓		
	
	QuickDASH	• Perception of abilities				✓
	
	OPUS QoL	• Quality of life				✓
	
	RHI	• Embodiment				✓

Functional	PSFS	• Perception of abilities				✓
	
	Modified UEFS	• Perception of abilities • Task willingness			✓	
	
	Foam block identification task	• Perception of abilities • Decision-making				✓

Usage	Onboard logs	• Duration of use • Active usage	*Continuously recorded*			

*The schedule for administering specific measures is indicated by the check marks.*

Sensation location, intensity, and quality were assessed twice daily throughout the study. The participant filled out surveys about each of the four sensations immediately after donning and calibrating the system and immediately prior to doffing the system. The participant reported the perceived sensation location for each sensor by outlining the location on a hand diagram (Tan et al., 2014). He reported the perceived intensity of the sensation and the degree to which several quality descriptor words (Supplementary Table S2) described the sensation using a series of visual-analog scales (VAS). The descriptors selected for this study were a subset of those presented in the prior home use study (Graczyk et al., 2018b) (descriptors that were never rated by the participant during the previous home trial were excluded here). The participant also reported the position of the phantom limb twice daily, prior to donning and doffing the system. The participant drew the perceived position of his phantom fingertips relative to their expected anatomic location on an arm diagram (Graczyk et al., 2018b).

Psychosocial outcomes were evaluated at two time scales. First, the participant completed the Take-Home Experience Diary (THED) in the evenings on days that he wore the system. The THED consisted of the short form of the Patient Experience Measure (PEM, see below) and free response questions to describe any notable experiences or circumstances that day (Graczyk et al., 2018b). During the monthly laboratory visits, the participant completed additional psychosocial surveys. The PEM consisted of five subscales including embodiment of the prosthesis, self-efficacy, social touch, body image, and prosthesis efficiency. The embodiment subscale measured the perception of ownership of the prosthesis (e.g., the prosthesis is a part of me), the self-efficacy subscale measured confidence in using the prosthesis for functional tasks, the social touch subscale measured the perceived ability to use the prosthesis in social situations (such as shaking hands), the body image subscale measured the impact of the prosthesis on the conscious perception of the body, and the prosthesis efficiency subscale measured the perceived speed and focus required to use the prosthesis (Graczyk et al., 2018b). Note that the short form of the PEM (administered daily) was an abridged version of the PEM and did not include the body image subscale. The Rubber Hand Illusion (RHI) questionnaire evaluated prosthesis embodiment immediately after performing a functional task in the laboratory setting (see below) (Ehrsson et al., 2008; Marasco et al., 2011; Schiefer et al., 2016). Two other surveys evaluated holistic psychosocial outcomes: the Orthotics and Prosthetics Users’ Survey (OPUS) Quality of life (QoL) metric was used to assess overall quality of life (Heinemann et al., 2003; Jarl et al., 2014), and the Quick Disabilities of the Arm, Shoulder and Hand (QuickDASH) survey evaluated the participant’s perception of his disability (Gummesson et al., 2006; Polson et al., 2010; Resnik and Borgia, 2015).

Functional outcomes were evaluated using surveys, standardized functional tests performed in the laboratory, and system logs. The participant’s ability to interpret sensory feedback was evaluated using the foam block task (Graczyk et al., 2018b; Schiefer et al., 2018). Briefly, the participant was asked to identify the size or compliance of foam blocks presented to the prosthesis without visual or auditory feedback. The blocks had three sizes (small, medium, large) or three compliances (soft, medium, hard). The participant completed the tasks both with and without sensory feedback during each laboratory visit. In addition to this objective measure of function, three measures were used to assess perceived function, which is the participant’s subjective view of their abilities with the prosthesis. Before each trial set of the foam block task, the participant was asked to report on his confidence in his ability to perform the upcoming foam block task (Graczyk et al., 2018b; Schiefer et al., 2018). Two standard clinical metrics were also used to evaluate perceived function. The Patient Specific Functional Scale (PSFS) required the subject to identify five tasks he had difficulty performing prior to the study, then tracked his perceived ability to perform those same tasks throughout the study (Stratford et al., 1995; Hefford et al., 2012; Resnik and Borgia, 2012). The modified OPUS Upper Extremity Functional Status (UEFS) survey evaluated the participant’s willingness and perceived ability to perform a set of 28 standard activities of daily living (ADL) (Heinemann et al., 2003; Jarl et al., 2012; Graczyk et al., 2018b).

Finally, the participant’s use of the sensory-enabled system was tracked through system logs. The system logs recorded system settings and button presses, such as enabling or disabling sensory feedback, and all activity from the prosthesis pressure and aperture sensors (Graczyk et al., 2018b).

### Qualitative Analysis

We conducted a qualitative analysis of in-person interview data to explore how the participant’s experiences with the sensory-enabled prosthesis changed over time. In each laboratory session, the participant completed a 20–40 min semi-structured interview with authors EG and IC. Interview questions explored the experience of sensation, the experience of the prosthesis, changes in sensation over time, and changes in prosthesis experience over time. See Supplementary Data Sheet S1 for interview questions. EG was the primary interviewer, while EG and IC both probed responses for clarification or expansion. The interview data were video recorded and transcribed by IC.

After completing the interviews, four of the investigators (IC, LR, AG, and EG) performed a modified grounded theory analysis using constant comparison methods (Strauss and Corbin, 1990, 1994; Creswell, 2007). We utilized the grounded theory approach to perform open coding, axial coding, and selective coding (Strauss and Corbin, 1998). NVivo 12 software was used to organize the data (NVivo qualitative data analysis Software; QSR International Pty Ltd., Version 12.3.0). In the open coding phase, the analysis team performed line-by-line open coding of the transcript from the interview at the end of interval 1. The investigators then established a preliminary set of codes through consensus coding (Haverkamp and Young, 2007). After creating the initial codebook, LR stepped away from the analytical discussions to serve as an external auditor of the analytic process and findings. Each successive interview transcript was then coded, and the code list and code definitions were iteratively fine-tuned through consensus of the analytic team (EG, AG, and IC). In the axial coding phase, the codes were separated into categories and sub-categories. Descriptions of each code were generated and supported with rich text exemplars. These code descriptions were then used to assist with the process of selective coding, in which the axial codes were organized into overarching themes. Throughout the analytic process, the team maintained an audit trail to track their decisions (Charmaz, 1996), and LR reviewed the audit trail.

The analysis was conducted from a primarily post-positivist epistemological framing, with some constructivist leanings (Mills et al., 2006; Creswell, 2007; Staller, 2013). Note that EG and IC are experts in neural engineering and sensory neuroprostheses, and each have over 5 years of experience working with this participant in associated research studies. LR and AG conduct research related to rehabilitation outcome assessment with a focus on upper limb prostheses, and have never met the participant. LR, EG, and AG had previous experience with grounded theory analysis of participant perspectives on sensory prostheses from the prior short-term home study (Graczyk et al., 2019), whereas IC did not.

### Statistical Analysis

Analysis was performed after all data was collected. Throughout, an alpha-value of 0.05 was used. All values are reported as mean ± standard error of the mean.

We studied both within-day and across-days changes in sensory perception (location, quality, and phantom limb length). Within-day changes were compared using paired *t*-tests. Across-day trends were evaluated using linear regressions over hours with sensation. Data for each sensory channel was only included for days on which its corresponding prosthesis sensor operated correctly, as determined by analysis of the onboard log and corroborated by participant daily diary reports. Specifically, data is shown for days 1–111 for channel 1, 1–36 for channel 2, 1–111 for channel 3, and 1–58 channel 4 (Supplementary Figure S1A). Analyses of within-day changes were performed only on days for which both morning (AM) and evening (PM) data is available. Across-day analyses include data from the morning surveys on days that sensors failed. Across-day analyses of location alignment are binned such that each point represents 5 days of collected data. However, the last point in each time series contains between 3 and 5 data points, based on the number of days the participant wore the system before the study ended. Additional statistical analyses for channels 1 and 4 are described in Supplementary Data Sheet S2. Further, two outliers, whose values were more than 3 standard deviations away from the mean, were removed from the analysis of the phantom limb length data.

Changes in psychosocial survey outcomes between in-lab sessions were performed by comparisons of successive data points. Surveys for which normative data exists (QuickDASH and OPUS QoL) were evaluated using their respective minimum detectible change values (MDC). The PEM was analyzed using paired *t*-tests of subscale items between successive in-lab sessions and between the first and last in-lab sessions. Agreement with embodiment vs. control statements in the RHI was analyzed using 2-sample *t*-tests, and trends across the study were analyzed using linear regression. Changes to the short form PEM scores over time were analyzed using linear regression.

Changes in functional outcomes over time, including foam block performance and confidence, modified UEFS task difficulty and completion rate, the PSFS, and active sensor usage, were evaluated using linear regression. As normative data exists for the PSFS, successive data points were also evaluated using its MDC.

Functional outcomes with and without sensation were also compared (see Supplementary Figure S2). The foam block test was evaluated both with and without sensation during this study, and comparisons were made using paired *t*-tests. In contrast, at-home measures, including the UEFS and active prosthesis usage, were always collected with sensation-enabled during this study. To compare performance with sensation vs. without sensation in these measures, data from this study, in which sensation was always enabled, was compared to sensation-disabled data from our previous study (Graczyk et al., 2018b). In addition, we compared sensation-enabled data from this study to the sensation-enabled data from the prior study. Statistical comparisons between the two studies were made using 1-way ANOVA followed by Tukey test.

## Results

The effects of learning on sensory perception (sensation location, sensation quality, and phantom limb) were evaluated within-day and across-day. Because we hypothesized that working prosthesis sensors were required for sensory learning, data for each sensory channel was only analyzed for days on which the sensor was known to have operated correctly, as determined by corroboration of the onboard log data with the participant’s daily diary. Note that channel 2 failed after 13 days of wear. The within-day comparison was used to study the immediate effects of actively using sensation on perception, whereas across-day trends over the 115-day study were examined to investigate whether any changes in sensation were retained over time. Across-day trends were evaluated independently from within-day changes, as we believe they may have different mechanisms or implications.

### Perceived Sensation Locations Become Aligned With Prosthesis Sensor Locations Over Time

**FIGURE 2:**
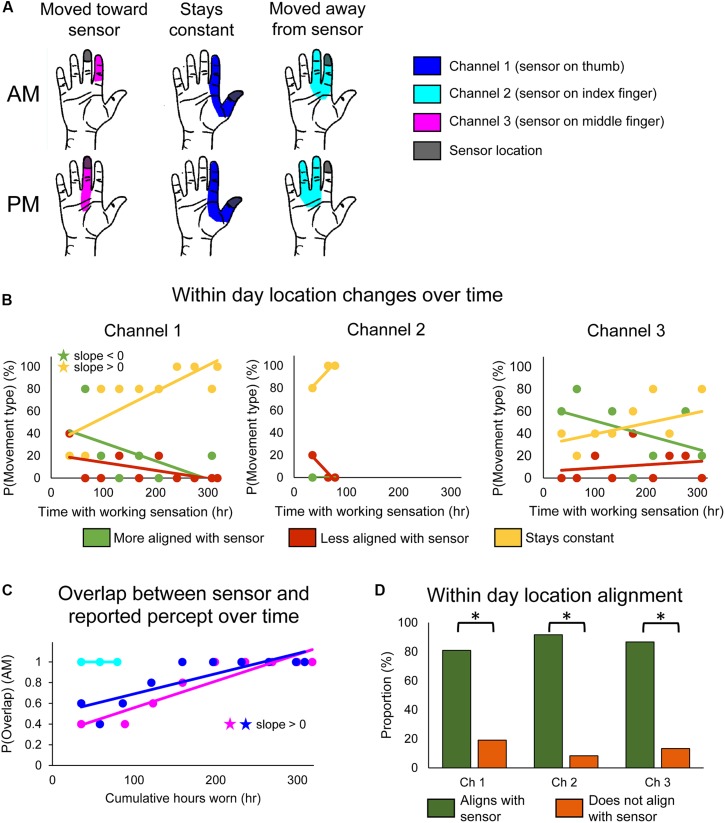
Changes in perceived sensory locations over the course of the study. **(A)** Examples of location shifts between donning (top) and doffing (bottom) the system in 1 day. The location could shift such that it became more aligned with the sensor location (left), stay constant (middle), or shift such that it became less aligned with the sensor location (right). **(B)** Scatter plots showing changes over the course of the study in the probability that the perceived sensation location would move toward the sensor (green), away from the sensor (red), or remain constant (yellow) over the course of a single day of functional use (*n* = 47, 12, 45 days of wear for channels 1, 2, and 3, respectively). **(C)** Scatter plot showing changes over the course of the study in the probability that perceived location would be aligned with the sensor location at the beginning of the day (AM), before any functional usage occurred (*n* = 49, 14, 48 days of wear for channels 1, 2, and 3, respectively). For **(B,C)**, stars denote statistically significant trends over time (*p* < 0.05). **(D)** Bar plot showing relative proportion of within-day movements that were aligned with sensor use (green) vs. not aligned with sensor use (orange) (*n* = 47, 12, 45 days of wear for channels 1, 2, and 3, respectively). Asterisks denote significant differences between groupings (*p* < 0.05).

We defined the perceived sensory location to be aligned with the prosthesis sensor if the location reported on the hand diagram overlapped the defined prosthesis sensor location. Within-day changes of sensory percept location were evaluated by comparing sensation locations between morning and evening drawings. We categorized sensations as either moving toward the sensor (Figure 2A, left), staying constant (Figure 2A, middle), or moving away from the sensor (Figure 2A, right) based on whether the drawings of the percept changed between morning and evening and whether the drawings overlapped with the prosthesis sensor position. Channel 4 was excluded from this analysis because the aperture sensor with which it was associated was mounted underneath the prosthesis cosmetic cover and thus did not have a defined location on the surface of the hand.

We found that stimulation-evoked sensation locations changed both within a single day of use and over the course of the study. There also were interactions between these two time courses: the types of within-day location changes exhibited across-day trends over the course of the study. For channel 1, the frequency at which the perceived sensation moved toward the sensor decreased over the course of the study (line slope test, *p* = 0.045) (Figure 2B, left, green), while the frequency at which perceived locations remained constant increased (line slope test, *p* = 0.006) (Figure 2B, left, yellow). Interestingly, there were no significant trends in sensations becoming misaligned with the sensor across the study (line slope test, *p* = 0.126) (Figure 2B, left, red). The reported sensation locations for channels 2 and 3 followed similar trends, although none were statistically significant (Figure 2B, middle and right).

These long-term trends of within-day change occurred as a result of the sensory alignment becoming more permanent through the study. To examine changes in sensation location across days, we tracked the proportion of days that the morning percept overlapped with the sensor position across the 115-day trial. Across days, the sensation location became significantly more frequently aligned with the sensor location upon donning the system (line slope test, *p* < 0.001 and *p* = 0.002 for channels 1 and 3, respectively) (Figure 2C). The perceived location for channel 2 was aligned with the sensor location throughout its brief period of functionality.

Throughout the study, the percept locations for the working sensors were more likely to be aligned with the sensor than not (test of 2-proportions, *p* < 0.001 for all channels) (Figure 2D). This was driven by within-day shifts toward the sensor at the beginning of the study and by the constantly aligned sensation later in the study. Interestingly, this trend is reversed for channel 2 on the days that the prosthesis sensor was malfunctioning (Supplementary Figure S1B). For channel 2, the percept location was significantly less likely to be aligned with the sensor when the sensor was malfunctioning than when it was working (test of 2-proportions, *p* = 0.001).

### Perceived Sensation Quality Became More Congruent With Transduced Information and More Natural Over Time

**FIGURE 3:**
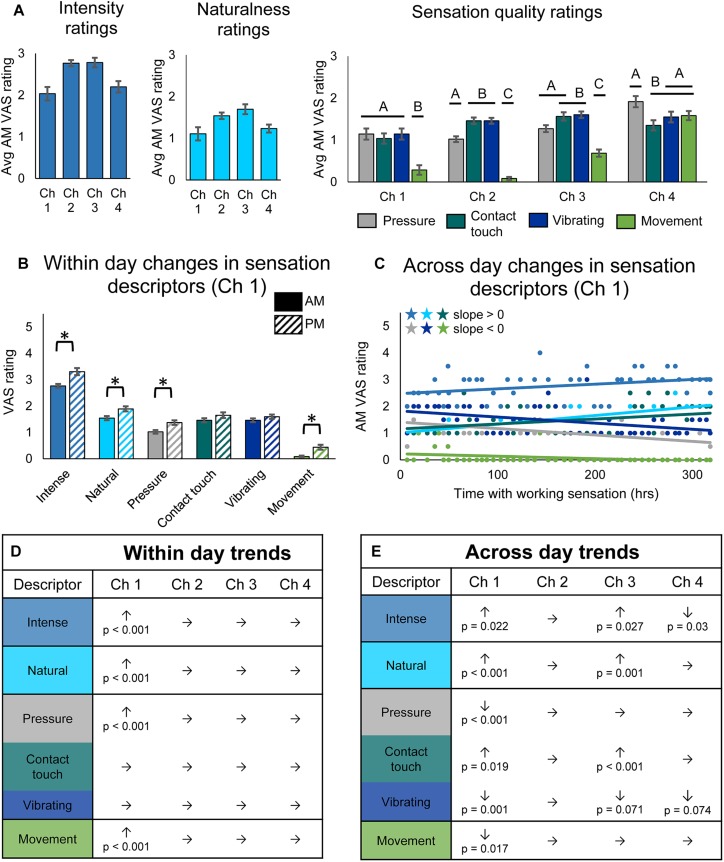
Changes in perceived sensation quality over the course of the study. **(A)** Average rating of quality descriptors upon donning the system for each channel (*n* = 49, 14, 48, 29 days of wear for channels 1, 2, 3, and 4, respectively). Horizontal bars denote statistical groupings based on a one-way ANOVA with Tukey pairwise comparisons. Bars that do not share a letter are significantly different (*p* < 0.05). **(B)** Bar plots depicting within-day changes between the morning (AM) and evening (PM) self-reported ratings of quality descriptor words for channel 1 (*n* = 47 days of wear). Asterisks denote significant differences between groupings (*p* < 0.05). **(C)** Scatter plot showing changes over the course of the study in self-reported ratings of quality descriptor words for channel 1 (*n* = 49). Descriptor words are color-coded as in the tables in **(D,E)**. Stars denote statistically significant trends over time (*p* < 0.05). **(D)** Table depicting within-day changes for all channels, based on paired *t*-tests comparing AM to PM ratings (*n* = 47, 12, 45, 28 days of wear for channels 1, 2, 3, and 4, respectively). Significant increases (↑) and decreases (↓) from AM to PM are shown with their corresponding *p*-values, and insignificant changes are indicated by (→). **(E)** Table depicting across-day changes for all channels, based on a linear regression of the AM descriptor ratings vs. hours with working sensation (*n* = 49, 14, 48, 29 days of wear for channels 1, 2, 3, and 4, respectively). Significant increases (↑) and decreases (↓) are shown with their corresponding *p*-value, and insignificant changes are indicated by (→).

The reported stimulation-evoked sensation qualities also changed over time. The subject rated the extent to which each of thirteen descriptor words characterized the perceived sensation on a VAS (Supplementary Table S2). The subject rated seven of the words, including “unpleasant,” “tingling,” “rough,” “electrical,” “sharp,” “cramping,” and “edged,” in less than 5% of the completed surveys, so these words were excluded from the analysis. It is interesting to note that these infrequently rated words are primarily qualities that could not be transduced by the simple sensors on the prosthesis, such as “rough,” or those with negative valence, such as “unpleasant.” Words that were rated in more than 5% of the surveys included “intense,” “natural,” “pressure,” “contact touch,” “vibrating,” and “movement.” For the purpose of this analysis, intensity is considered a dimension of sensation orthogonal to quality (Figure 3A, left), and natural is considered a judgment about the holistic experience of sensation (Figure 3A, middle). The other four descriptors can be categorized as tactile (“pressure,” “contact touch,” and “vibrating”) or proprioceptive (“movement”) (Figure 3A, right). Based on the definitions provided to the participant (Supplementary Table S2), “pressure” was associated with compression or weight, while “contact touch” involved light touch sensation from a voluntary action. It is interesting to note that the participant consistently rated the proprioceptive descriptor significantly lower than the tactile descriptors for the tactile channels 1–3 (1-way ANOVA followed by Tukey, *p* < 0.001 for all three channels), but rated the proprioceptive descriptor equivalently to the tactile descriptors for channel 4, which was associated with the aperture sensor.

As with sensory percept location, we examined within-day (Figure 3B) and across-day changes (Figure 3C) in sensory percept intensity, naturalness, and quality. There were no significant within-day changes in either sensation intensity, naturalness, or quality for channels 2, 3, and 4, but there were significant changes for channel 1 (Figures 3B,D). For channel 1, the intensity, naturalness, pressure, and movement of the sensation all significantly increased within a day of usage (paired *t*-test, *p* < 0.001).

Across-day changes in the sensation intensity, quality, and naturalness over the course of the study were more prevalent across channels (Figures 3C,E). The reported intensity of the percepts increased across the 115-day study for two of the tactile channels (line slope test, *p* = 0.022 and *p* = 0.027 for channels 1 and 3, respectively) and decreased for the proprioceptive channel (line slope test, *p* = 0.03). The participant also reported increases in naturalness for the two working tactile channels (line slope test, *p* < 0.001 and *p* = 0.001, for channels 1 and 3, respectively). Quality descriptors also changed over the duration of the study. Ratings of contact touch significantly increased throughout the study for the working tactile sensors (line slope test, *p* = 0.019 and *p* < 0.001, for channels 1 and 3, respectively). The contact touch descriptor is likely most similar to the information transduced by the pressure sensors on the prosthesis during functional use. Conversely, the participant’s ratings of words that did not align with the use cases of the tactile sensors, including pressure, vibrating, and movement, either decreased or did not significantly change (line slope test, channel 1: *p* < 0.001, *p* < 0.001, and *p* = 0.017, respectively; channel 3: *p* = 0.73, *p* < 0.001, and *p* = 0.071, respectively) over the course of the study. The across-day changes in sensation naturalness and quality reflect increased congruency with transduced sensor information (Figure 3E).

Throughout the study, there were no significant trends for channel 2, the non-working tactile sensor. Unlike the perceived sensation location, there also were no significant differences in the reported VAS ratings for descriptor words for days when the sensor was working vs. non-working (Supplementary Figure S1C).

Quality and naturalness ratings for channel 4, which was associated with the prosthesis aperture sensor, did not change over time, although this may be an artifact of the lower sample size due to only analyzing data from intervals 1 and 2 of the study. Indeed, analysis of all three intervals (see Supplementary Data Sheet S2) shows significant trends in four of the descriptor words (Supplementary Figure S1E). Over the full 115-day study, both intensity and naturalness significantly increased for channel 4 (linear regression, *p* = 0.001 and *p* < 0.001, respectively), as they did for the two working tactile channels. The participant’s ratings of tactile descriptors did not trend together: his rating of “pressure” increased although not significantly (linear regression, *p* = 0.072), his rating of “contact touch” significantly increased (linear regression, *p* = 0.006), and his rating of vibration significantly decreased (linear regression, *p* = 0.006). Surprisingly, the participant’s rating of the proprioceptive descriptor, “movement,” which trended upward, did not significantly increase over the course of the study (linear regression, *p* = 0.162). There were no significant differences in within-day changes for channel 4, even when analyzing the full data set (Supplementary Figure S1D).

### Perceived Phantom Position Aligned With the Prosthesis Over Time

**FIGURE 4:**
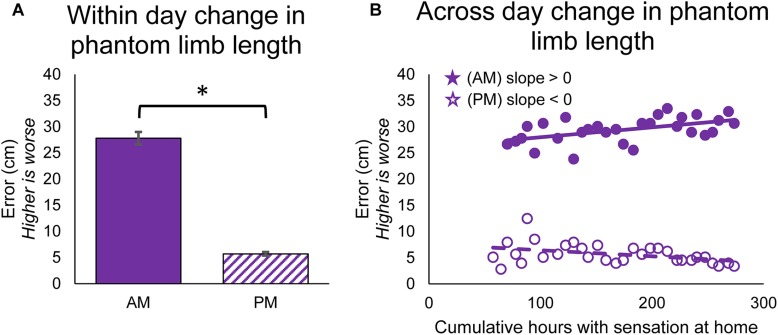
Changes in perceived position of the phantom limb over the course of the study. **(A)** Average error between phantom limb location and prosthesis location in the mornings (AM) and evenings (PM). Higher error indicates that the phantom limb was more retracted into the residual limb (telescoped) and lower error indicates that the phantom limb was more extended toward an anatomically-appropriate position. Asterisks denote significant differences between groupings (*p* < 0.05). **(B)** Scatter plot showing changes over the course of the study in the error between phantom limb location and prosthesis location in the mornings and evenings (*n* = 31 for AM and PM). Stars denote statistically significant trends over time (*p* < 0.05).

The position of the phantom limb also changed over time. The participant reported the position of the phantom fingertips relative to the position of the prosthesis fingertips using a drawing, and the error in phantom limb length was measured. The phantom limb became significantly more aligned with the location of the prosthetic hand within-days (paired *t*-test, *p* < 0.001) (Figure 4A). The alignment of the phantom limb with the prosthetic hand also improved across-days for the evening drawings, as shown by the decrease in limb length error over time (line slope test, *p* = 0.026) (Figure 4B, open circles). However, the within-day improvements in alignment did not carry over to the next morning (Figure 4B, filled circles). In fact, the phantom limb length error increased over time for the morning drawings (line slope test, *p* = 0.005).

### Psychosocial and Functional Outcomes Improve With Use of a Sensory Prosthesis

**FIGURE 5:**
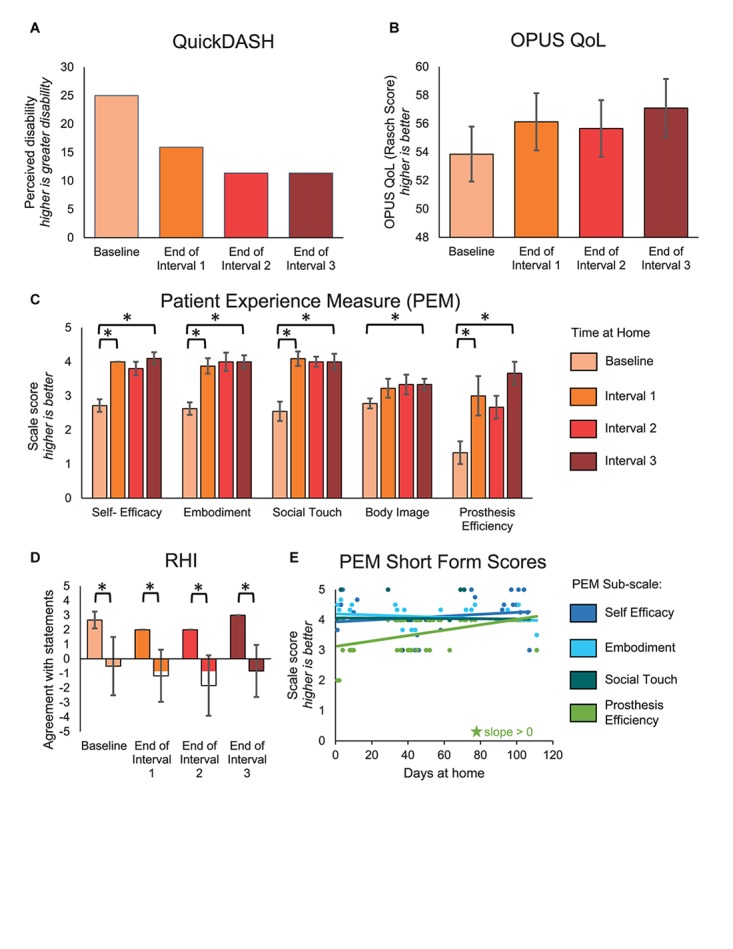
Psychosocial outcomes over the course of the study. **(A–D)** The QuickDASH, OPUS QoL, PEM, and RHI were administered prior to the start of the study and then monthly during the participant’s laboratory visits. For the QuickDASH, OPUS QoL, and PEM, comparisons were made between the beginning and end of the study and between successive intervals of the study. For the RHI, comparisons were made between embodiment and control statements within each session. **(A)** The QuickDASH is a measure of the participant’s perceived disability. For this measure, a higher score indicates a worse outcome (*n* = 4 scores). **(B)** The OPUS QoL is a holistic measure of the participant’s quality of life (*n* = 4 scores). A higher score indicates a better quality of life. **(C)** The PEM measures the participant’s perception of various psychosocial outcomes (*n* = 10, 8, 11, 9, 3 items in the self-efficacy, embodiment, social touch, body image, and prosthesis efficiency sub-scales, respectively). For each sub-scale, a higher score indicates a better outcome. **(D)** The RHI survey is a measure of the extent to which the participant embodied the sensory-enabled prosthesis (*n* = 3, 6 items in the embodiment and control groups, respectively). Positive values indicate more agreement with the survey statements and negative values indicate more disagreement with the survey statements. For **(A–D)**, asterisks denote significant differences between groupings (*p* < 0.05). **(E)** A short form of the PEM was administered daily at-home (*n* = 48 days of wear). Longitudinal trends were analyzed. Stars denote statistically significant trends over time (*p* < 0.05).

Beyond changes in sensory perception, we also investigated the effects of long-term usage of a sensory enabled prosthesis on psychosocial and functional outcomes. The QuickDASH and OPUS QoL are holistic measures of the subject’s perceived disability and quality of life, respectively. These surveys trended toward improvement but did not reach significance. The participant’s score on the QuickDASH improved by 13.6 points over the course of the study [MDC-95 = 17.4 (Resnik and Borgia, 2015)] (Figure 5A). Similarly, the participant’s score on the OPUS QoL improved by 3.24 points over the course of the study [MDC-95 = 7.4 (Jarl et al., 2014)] (Figure 5B).

The participant had significant improvements in several subscales of the PEM (Figure 5C). The participant’s ratings on the self-efficacy, embodiment, and social touch subscales all significantly improved within the first month (paired *t*-test, *p* = 0.001, *p* = 0.005, and *p* < 0.001, respectively), then stabilized. Prosthesis efficiency also significantly improved within the first interval (paired *t*-test, *p* = 0.038), but then continued improving through the remaining intervals. In contrast, the participant’s ratings on the body image subscale did not improve significantly within any single interval but did improve significantly over the entire study (paired *t*-test, *p* = 0.013). The improvement in embodiment shown by the PEM was supported by the RHI (Figure 5D). While the RHI scores did not significantly improve over time (line slope test, *p* = 0.593), the participant did significantly embody his sensory-enabled prosthesis throughout the study, as evidenced by the significant difference between the embodiment and control statements (two-sample *t*-test, *p* ≤ 0.015). Interestingly, the short form of the PEM, which was administered daily, only showed a significant increase in the prosthesis efficiency subscale over time (line slope test, *p* < 0.001) (Figure 5E). There were no other significant trends over time on the PEM short form.

**FIGURE 6:**
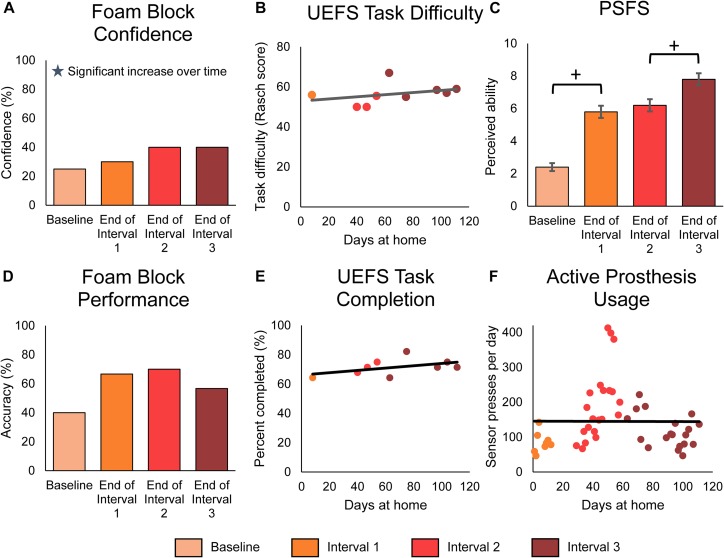
Functional outcomes over the course of the study. Panels **(A–C)** demonstrate the participant’s perceived functional ability with the prosthesis, while panel **(D)** demonstrates the participant’s actual functional ability. Panels **(E,F)** demonstrate the participant’s active engagement of the prosthesis in functional tasks. For all panels, stars denote statistically significant trends over time (*p* < 0.05). **(A)** The participant’s confidence in performing the foam block task significantly increased over time. The foam block measure was administered prior to the start of the study and then approximately monthly during the participant’s laboratory visits (*n* = 4 scores). **(B)** The participant’s rating of difficulty in performing tasks with the prosthesis, as measured by the modified UEFS. The modified UEFS was administered approximately weekly at-home (*n* = 9 scores). **(C)** The PSFS measured the participant’s perceived ability to do a set of functional activities chosen by the participant. The PSFS was administered prior to the start of the study and then approximately monthly during the participant’s visits (*n* = 4 scores). Crosses denote significant differences between groupings based on MDC-95 values. **(D)** The foam block task objectively measured the participant’s ability to identify objects with the prosthesis during laboratory sessions (*n* = 4 scores). **(E)** The proportion of tasks on the modified UEFS that the participant reported completing each week with the prosthesis (*n* = 9 scores). **(F)** The participant’s active usage of the prosthesis at-home was measured by the number of presses on the prosthesis sensors logged by the system each day (*n* = 48 days of wear).

In addition, we examined changes in functional outcomes over the course of the study. We found that the participant’s perceived ability to use his prosthesis for functional tasks improved over time. First, we evaluated the participant’s confidence in his ability to identify the size or compliance of a foam block using his prosthesis without visual or auditory feedback. As previously reported (Graczyk et al., 2018b; Schiefer et al., 2018), he was significantly more confident in performing this laboratory task when using the prosthesis with sensation compared to without (paired *t*-test, *p* < 0.001) (Supplementary Figure S2A). Further, his confidence in performing this laboratory task significantly improved over the duration of the study (Figure 6A, line slope test *p* = 0.011). Second, the participant reported his perceived ability to do standard ADLs and self-identified, personally relevant tasks at-home through the modified OPUS UEFS difficulty rating and the PSFS, respectively. The modified OPUS UEFS difficulty rating did not improve with sensation (2-sample *t*-test *p* = 0.584) (Supplementary Figure S2C) or over time (line slope test, 0.355) (Figure 6B). However, his ratings on the PSFS improved significantly across the duration of the study (MDC-95 = 1.3, total improvement = 5.4 points) (Figure 6C). The most dramatic improvement in PSFS scores occurred within the first month (3.4 points), but continued to improve significantly within the last month of the study (1.6 points). The participant’s self-identified tasks in the PSFS included peeling vegetables, cutting food and meal preparation, holding someone’s hand, folding clothes, and putting away dishes.

Surprisingly, the participant’s actual ability to perform functional tasks with the sensory-enabled prosthesis, as measured by laboratory performance of the foam block test, did not improve over time (line slope test *p* = 0.562) (Figure 6D). However, as with the participant’s confidence in the task, his objective ability to perform the task was significantly better with sensation compared to without (Supplementary Figure S2B, paired *t*-test *p* = 0.003).

Finally, we measured the participant’s engagement with the prosthesis using the modified OPUS UEFS completion rate and daily sensor presses. The modified OPUS UEFS completion rate assessed how many tasks out of the 28-task UEFS list the participant chose to attempt each day. The daily sensor presses indicated how many times the participant actively interacted with the prosthesis by pressing on the sensors or performing a grasp with the prosthesis. Neither of these measures significantly increased over the course of the study (line slope test, *p* = 0.199 and *p* = 0.975, respectively) (Figures 6E,F). However, the participant did use the sensory-enabled prosthesis significantly more actively than a standard prosthesis, as indicated by the increase in modified UEFS tasks completed and sensor presses compared to data without sensation from a previous study (2-sample *t*-test, *p* < 0.001 in both) (Supplementary Figures S2D,E).

### Qualitative Findings of Sensation Experience, Prosthesis Engagement, and Embodiment Change Over Time

The qualitative analysis resulted in the identification of four primary themes: sensation experience, learning, prosthesis engagement, and embodiment. The sub-categories within each theme and a brief description of each is provided in Table 2. We present each of the themes below with rich text exemplars. Brackets denote interjected text to improve readability, and parentheses denote gestures. Each exemplar’s transcript number is indicated at the end of the quotation.

**TABLE 2:** Primary themes and associated sub-categories generated through the qualitative analysis.

**Theme**	**Sub-categories**	**Brief description of sub-category**
Sensation experience	Sensation description	Comments about the location, intensity, modality, or quality of sensation
	
	Stereognosis	Description of how sensory feedback provided holistic feedback about object interactions or physical object features (such as object shape)
	
	Preference for sensation	Comments about the subject’s views (positive or negative) about sensory feedback

Learning	Usefulness of sensation	Description of how sensory feedback was helpful in performing tasks or interacting with others
	
	Mechanisms of learning	Description of how the participant improved his ability to use or interpret sensation
	
	Ease and attention	Comments about the effort and/or attention required to use the prosthesis

Prosthesis engagement	Functional tasks	Comments about performing functional tasks with the prosthesis
	Bilateral activities	Comments about using the prosthesis in conjunction with the intact hand to perform bilateral activities
	
	Interaction with others	Comments about using the prosthesis in social interactions
	
	Confidence	Comments about the participant’s perception of his ability to successfully use the sensory prosthesis

Embodiment	My hand	Comments showing ownership of the prosthesis as belonging to the body or incorporation into his body representation
	
	Naturalness	Comments about the normalcy of the sensation experience or the sensory prosthesis
	
	Perception of phantom limb	Description of his phantom limb position relative to the prosthesis location

All themes were modulated by time, in that continued exposure to the sensory feedback and extended usage of the sensory-enabled prosthesis led to long-term changes in experiences. The qualitative analysis also produced a secondary theme, system operation, which is described in Supplementary Data Sheet S3. This theme included comments related to technical functionality of system components, and as it did not change over time, it did not address our primary research question of long-term changes in sensation or experience. Full definitions for all themes and sub-categories are provided in Supplementary Table S3.

**FIGURE 7:**
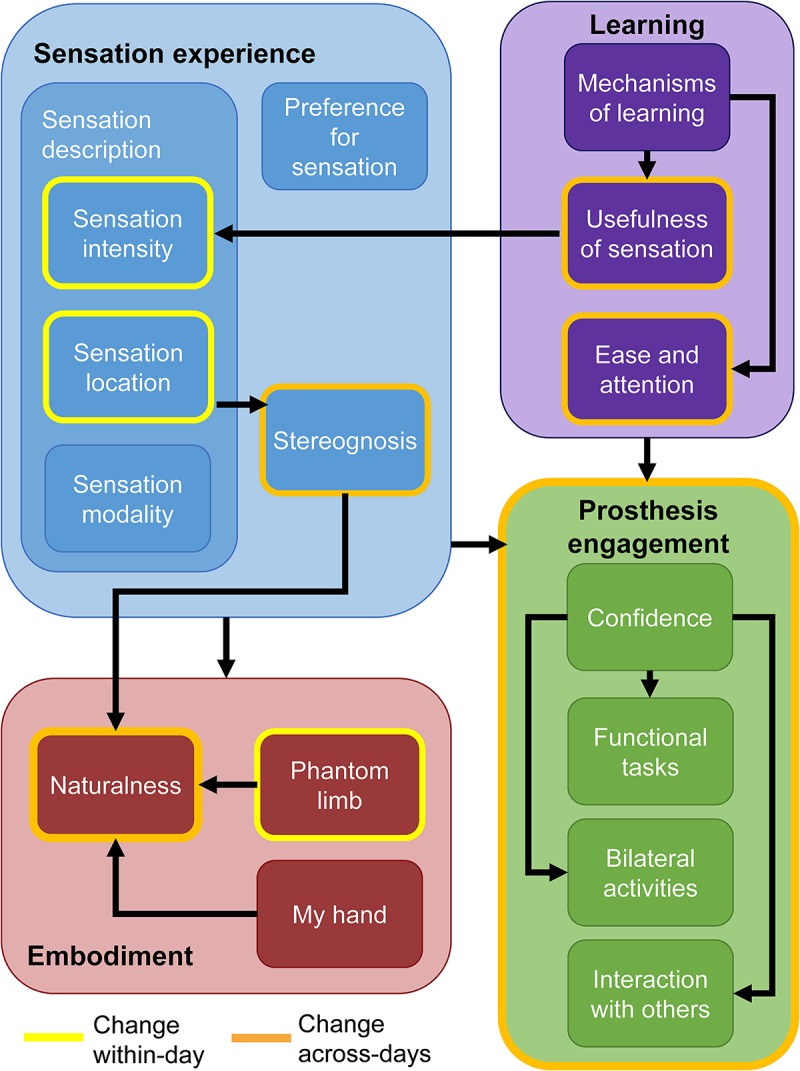
Findings from the qualitative analysis. Themes and their associated sub-categories are indicated by fill color. Categories outlined in yellow are those which the participant noted changed within individual days. Categories and themes outlined in orange changed over the course of the study. Black arrows indicate tentative relationships among themes and sub-categories. Having sensory feedback in the prosthesis contributed to increases in all sub-categories within the embodiment and prosthesis engagement themes.

In short, the participant described key changes in his experience over time within the four primary themes (Figure 7). These changes occurred either within-days or across the duration of the study. Within individual days, the sensation intensity became stronger, the sensation location became more focal, and the phantom limb became better aligned with the prosthesis. Over the course of the study, the two most prominent changes were increases in the naturalness of the experience and increases in prosthesis engagement. The naturalness of the experience included both the sensation experience and the prosthesis experience. In addition, over time, his perception of the sensation experience began to include perceptions of stereognosis. Stereognosis is defined as “the ability to perceive the form of an object by using the sense of touch” and includes the perception of object features such as shape, size, and weight (Carlson and Brooks, 2009). The increase in prosthesis engagement was exemplified by the participant’s reported increased willingness to do functional and bilateral tasks with the prosthesis and to use the prosthesis in social interactions. Through both passive and active learning mechanisms, the sensations became more useful in accomplishing tasks over time and the ease of using the prosthesis increased. Throughout the study, the participant described his preference for sensation and demonstrated embodiment of the prosthesis.

#### Sensation Experience

The sensation experience theme explains how the participant perceived and interacted with sensation over the course of the study. The participant described three dimensions of sensation – intensity, location, and quality. He described the intensity of the sensation as providing information about his grip strength with the prosthesis.

*“The stronger it gets, along with the pressure and everything, tells me how hard I’m touching something.”* [T3]

The participant also described how perceived sensation location provided information about which prosthesis sensor was engaged.

*“When I press [the sensors] individually they still feel the individual spots and everything … according to which one I press on, where sensation is at.”* [T2]

He stated that the intensity of sensation would generally increase and that the location would sometimes become more focal over a day of use.

*“Usually intensity just gets stronger throughout the day.”* [T2]

*“Some days it would localize a little more, like umm sometimes in the morning it’d feel like it was index and the thumb and later in the day or when I’d take it off and do the paperwork in the evening, it felt like it was localized in just like the index finger.”* [T1]

Interestingly, he did not report across-day changes in sensation type or location over the duration of the study. However, he did report that he was learning how to understand the sensations better with time.

*“Not really different every day because they’re pretty much in the same place, same sensation every day. So it’s just a matter of learning how to try to hone it in better.”* [T2]

The participant reported that the sensory experience became more natural over the course of the study.

*“It just felt more natural, like you were actually grabbing and holding something like that (picks up water bottle with intact hand and transfers to prosthesis to hold).”* [T3]

In later interviews, the participant began describing how the individual sensations provided by the sensors began to “work together” such that the sensory experience became more holistic and complete.

*“It felt more like one large global, it just felt like [the sensations] were all working together, merging together. It was my hand grabbing it, not just these fingers and then I could feel the other. It just kinda, all was kinda together.”* [T2]

Further, he indicated that he began to have the experience of holding an object in his hand (stereognosis), because the sensation experience reflected the shape of the object he grasped.

*“… it feels like (grasps water bottle with prosthesis) something’s right in that area right through there (gestures at thumb through index finger). So I can feel the light pressure, the vibration and everything and it- it just- some of it just feels natural because it feels like (gestures at prosthesis) something is right there, that shape, that you’re grabbing. Which a lot of times it’s something round I’m grabbing anyway, so that’s what it feels like. Holding a cup? I’m holding a cup.”* [T3]

The sensory feedback altered his perceived ability to do tasks and his ability to engage in social interactions.

*“So, that’s one of the best experiences with it, is being able to play with them [grandchildren] and know that I’m not hurting them. I’m grabbing and squeezing, and I can tell how hard I’m holding them and things. Umm, we’ve been out places, and people come up and shake hands and stuff.”* [T3]

As a result of having the sensory feedback, the participant was more willing to use his prosthesis rather than only relying on his intact, left hand.

*“It’s just umm, it’s [the sensation] helped me to start using my right arm more than what I have been and everything with the prosthetic on, because I can actually feel when I grab and touch stuff.”* [T3]

The participant reported an overall positive experience with sensory feedback. He frequently reported that the “sensory feedback was good” [T3] and that he “liked feeling my hand” [T2]. He reported a preference for having sensory feedback, saying:

*“I prefer having the sensory feedback. It gives me that feedback I need to know what I’m doing with that [prosthetic] hand, and how hard I’m squeezing on things, how hard I’m touching people. And the feedback’s just great.”* [T1]

At the end of the study, the participant affirmed that he would like to wear the sensory feedback system again in the future, stating:

*“Oh yeah. Yeah. Def-def- definitely. Because, like I said, I feel better with the sensation than without it.”* [T3]

Although the participant experienced several extended illnesses and breakages of components that interrupted his study, he explained that these problems did not change his desire to have the sensory feedback.

“I mean, yeah, it’d be nice if that all worked right, but I still prefer having [sensory feedback] as not having it.”

#### Learning

The theme of learning explains how the participant improved his ability to use his sensory-enabled prosthesis over the course of the study. When asked if he felt like he was learning to use the sensory feedback, he replied:

*“That’s the perfect way to put it. Umm, gaining skills, umm learning how the sensation, basically, which sensation is. How strong, how weak, and those sort of things. To make me use it even better.”* [T2]

Throughout the study, the participant described sensory feedback as being useful for performing functional tasks or interacting with others. He predominantly described using the sensation intensity to regulate his grip force:

*“I could tell if it [sensation] started to lighten up to squeeze a little harder and everything and hold [the dishes] while I opened the cabinet door (mimes opening an overhead cabinet with intact arm) and reach up and put them in (mimes bimanually putting plate in overhead cabinet) and all.”* [T3]

He learned to interpret the intensity of the sensation as related to his applied grip strength, saying:

*“But I can do it real light like that (gently pinches index sensor, then middle finger sensor), so it feels like that, or I can press real hard (increases pressure on middle finger sensor)… I’ll do it to umm feel the difference in the pressure, to feel how hard I’m grabbing things, to know how hard I’m grabbing things or how lightly I’m grabbing them.”* [T3]

He described the process of learning to use sensory feedback as happening both passively and actively. In passive learning, the improvements in sensory feedback discrimination occurred through exposure to the sensory feedback over time.

*“The more I wear [the prosthesis] with the sensory feedback, the more feedback I get, the easier it is to use, the better it is to use, tell the difference in the sensation or the pressure.’*’

In active learning, the participant described how he purposefully practiced receiving different levels of sensory feedback and interpreting it correctly as grip strength.

*“And then there’s times, like I say, I just sit down and want to feel my hand so I’ll do like this (squeezes on intact hand with prosthesis). How light can I go? How strong can I go? And work on feeling the difference.”* [T2]

*“A lot of it was practice … Pressing and start squeezing to feel how strong it would go, how light I could get it and that (repeats squeezing motion on sensors). That’s where a lot of it, for me, came from and everything. And actually doing it (makes reach and grasp motion with prosthesis). Actually grabbing things.”* [T3]

As he practiced interpreting the sensation, he described improvements in several hallmarks of learning. He described an improved ability to perform tasks with a decrease in errors or accidents:

*“And umm less apt to have those accidents of dropping something with this (makes grasping motion with prosthesis), because I feel [the sensation] start to lighten up a little bit (mimes startle reflex – twitches shoulders up) and I’ll squeeze back tight again and all. So that’s one difference.”* [T3]

As another indicator of learning, he described decreases in effort to perform the task:

*“I got a light grip there to a heavy grip there. And learning the difference in that. Which, you need to think about it a little bit, but more I wear it, the less I have to think about them. the more it becomes natural.”* [T2]

*“I can tell where I’m at and what I need to and I can hold it there. It’s just easier with the sensory cause I don’t have to watch it as close.”* [T2]

#### Prosthesis Engagement

The theme of prosthesis engagement addresses the participant’s views on his usage of the prosthesis and the extent to which he wanted to or was willing to wear and use the prosthesis. The participant described how having sensory feedback made him want to wear and use the prosthesis more. Prior to the study, he described a lack of confidence in his standard device:

*“Just grabbing dishes and putting them up in the cabinets and everything with my left [intact] hand a lot of times and not the right because it was just easier, faster, and didn’t feel as confident*…*”* [T1]

When asked if sensation changed the way he used the prosthesis, he said:

*“I started using it more and started wearing it more.”* [T2]

The participant described certain tasks that he was more likely to do or try now that he had sensation in the prosthesis.

*“I was more apt to grab ahold the grandkids and grab other things around the house that I normally don’t and was getting to where I’m using the hand more and more than what I have in the past years without sensation, and wearing it a lot more.”* [T2]

Having sensation also motivated the participant to want to engage the prosthesis more actively in everyday tasks. Instead of using only his intact hand for tasks, he was more willing to try tasks with the prosthesis.

*“It’s just starting to feel more normal to me, and using it more often. Using it to try to do things, umm, using it to try to grab dishes out of the dishwasher, plates and that kind of stuff, and put away rather than using my one – doing my one hand. Used to be, even when I had it on, I’d, I don’t know why, I’d just hold back on it, and grab with my good hand. Now, cause I can feel, I mean, I have a tendency to use it more.”* [T2]

In addition to using the prosthetic hand more frequently in skilled unilateral activities, the participant was also more willing to use the prosthesis in bilateral activities.

*“I’m more apt to put a knife in here (points to prosthesis). Or a fork in here and hold the fork and cut my food up and stuff with the knife and all. Umm even being able to hold a potato and tell that I’m holding it and how hard I’m squeezing it while trying to peel a potato (mimes peeling a potato while holding the potato in the prosthesis and a peeler in the intact hand) and stuff. It’s all different when you can feel it and when you can’t.”* [T3]

He also was more likely to use the prosthesis to interact with other people, such as when shaking hands.

*“We’ve been out places, and people come up and shake hands and stuff. And that’s a big difference too because used to be, it’d be my left hand (gestures with intact hand), and now I’m more apt to put my right arm out there (gestures with prosthesis) to shake somebody’s hand.”* [T3]

His willingness to use the prosthesis more actively appeared to change over the course of the study.

*“The more I wear the system, the more I use my right hand to help the left hand, whereas before I used to just keep it off to the side more.”* [T1]

Using the prosthesis more also helped him to learn how to interpret the sensory feedback, which then led to him wanting to use the prosthesis more.

*“A lot of it was practice just like this (squeezes repeatedly on thumb sensor)… Actually grabbing things, and just make myself use it more. And then want to use it more.”* [T3]

The participant also explained how his willingness to wear the prosthesis increased across months of the home study. He described this change as being directly related to his increased confidence in using the device correctly with sensory feedback:

*“I would use the hand even more this past month than what I did the previous month to do stuff and everything with the sensation and all. Because, confidence was building. It was becoming more normal.”* [T3]

#### Embodiment

The theme of embodiment addresses concepts related to limb ownership, body schema, body image, and perception of the phantom limb. The participant reported that the sensory experience facilitated his experience of device embodiment, and that both of these factors contributed to his prosthesis experience becoming more natural over time.

The prosthesis felt more like a part of his body when the participant had sensation, as if it were his actual hand rather than a tool.

*“Well, it feels more like it’s my hand and more like a part of me than just a tool that I’m using.”* [T1]

The participant expressed ownership of the prosthesis as if it belonged to his body. The prosthesis was viewed as being part of the body even though its attachment to his body was not permanent.

*“Yeah, cuz, view it* [the prosthesis] *more of being a part of me. Me, my limb, even though I take it off at night and everything, still being my limb when I’m wearing it. Because I can feel what I’m doing with it, or at least tell that I’m touching things.”* [T2]

The perception that the prosthesis was a part of him was associated with the position of his phantom hand. The participant explained that, when he had sensation, the position of his perceived phantom became more congruent with the prosthesis position. It appears that the sensory feedback led to decreases in limb telescoping, as the length of his phantom limb began to extend toward the fingertips of the prosthetic device.

*“Yeah, because it feels more like my hand and not just a tool extended from my arm. Because I can actually feel it. And, wearing it with the sensation actually, proprioception is putting it right about where the hand is at (gestures with prosthesis) with the prosthetic. It feels like, my fingertips feel like they’re about right in here (points to distal interphalangeal joint of prosthetic fingers), which is pretty close to where they are. Umm, when I’m not wearing [the prosthesis], getting up in the mornings and everything, and I think about [the phantom], then it feels like it’s back where it is (gestures to residual limb)… I think it kinda changes when I start feeling the sensation in the fingers (grasps thumb sensor with intact hand) when I put it on and start calibrating it in the morning. For some reason, it just feels like that’s where it’s at, and not back where I’m amputated at (gestures at residual limb).”* [T3]

The experience of embodiment was interconnected with the experience of normalcy or naturalness of the prosthesis.

*“I mean, like I say, when you got the sensation it just it- it feels like my hand, so it feels more natural.”* [T3]

The naturalness of the experience was also related to the position of the phantom limb. When asked to define the way he was using the word “natural” in the interview, the participant explained:

*“It feels like my hand’s actually there, and it’s not just, like I said, a tool added on. It umm brings the sensation out to where my hand was, in the fingers, wearing it and all. And it gives me sensation of my fingers that I can feel.”* [T3]

The participant remarked repeatedly that *“the more I have the system on and wear it, the more natural it becomes to me”* [T3]. In fact, the participant reported on the increase in naturalness or normalcy of the sensation over time at least a dozen times throughout the interviews.

*“The more I used it, the more natural, more or less, it became to me. The more, felt more normal, not necessarily like my left hand, but more normal to me.”* [T1]

The participant also described how the sensation experience became more natural throughout the study.

*“[The sensations] just felt more natural to me. Sometimes it felt more like pressure than necessarily the vibration and sometimes movement and everything. And it just, like I said, felt like somebody squeezing on those three fingers or just grabbing and holding them or like holding something in that area (gestures at water bottle).”* [T2]

In the third month, the participant described that, although the sensation itself wasn’t changing, the naturalness of the experience continued to improve.

*“Sensation didn’t really change other than the fact that, to me, it just became more and more normal and natural.”* [T3]

## Discussion

The participant in this case study used the sensory-enabled prosthesis for a total of 49 days over the course of the 115-day study and reported overall positive experiences with the sensory restoration system at-home. To our knowledge, this is the longest home use trial of a sensory-enabled prosthesis and is also the first study to examine the effects of learning on artificial sensation produced by electrical stimulation of the peripheral nerves. While the participant was not blinded to the presence of sensation over the course of the study, he was blinded to the objectives and hypotheses of the study in order to limit potential biases in his responses. In this study, we showed that perception of both evoked sensations and the phantom limb changed with prolonged exposure to sensory stimulation to become more congruent with the information provided through prosthesis use. Further, we found that psychosocial survey scores of self-efficacy, prosthesis embodiment, body image, social touch, and prosthesis efficiency were significantly higher while using the sensory feedback prosthesis at-home than at pre-test. Finally, we showed that perceived function significantly improved with sensation and usage. These findings were corroborated by the outcomes from the qualitative analysis, which described the subjective experiences of sensation, learning, prosthesis engagement, and embodiment. While limited conclusions can be drawn about generalizability from this case study, our findings agree with results reported in the sensory substitution (Dietrich et al., 2012, 2018) and cochlear prosthesis literature (Fu and Galvin, 2007, 2012), lending credence to our results. Our findings suggest that both passive and active learning modulate the perceptual and psychological experience of a sensory-enabled prosthesis over time.

### Implications of Changes in Sensory Perception Over Time

The participant reported changes in the sensory percepts produced by stimulation both within single days of use and across the duration of the study. Within-days, the perceived sensations changed to become more congruent with transduced sensor information. Several cognitive processes may drive these observed short-term sensory changes. In the intact system, information acquired from multiple sensory modalities, such as vision and touch, is integrated to form global percepts of the environment or specific objects within the environ- ment (Ernst and Bülthoff, 2004; Lederman and Klatzky, 2009; van Atteveldt et al., 2014). When there are significant discrepancies in information between modalities, tactile perception is momentarily altered such that it aligns with the presented visual information in a phenomenon known as visual capture (Lederman et al., 1986; Shimojou, 1987; Pavani et al., 2000; Ernst and Banks, 2002; Deneve and Pouget, 2004; Kersten et al., 2004; Körding et al., 2007; Gallace and Spence, 2008; Giummarra et al., 2008; Stanford et al., 2008; Lederman and Klatzky, 2009; van Atteveldt et al., 2014). When grasping objects with the prosthesis, the participant would have received visual cues about how the prosthesis sensors were contacted while feeling somatosensory feedback. Thus, multi-sensory integration and visual capture likely influenced the participant’s tactile perception during object interactions.

However, visual cues were minimized when the participant was completing the morning and evening surveys about his sensory experience, because he directly activated sensation through button presses on the neurostimulator rather than triggering sensation via the prosthesis sensors. Thus, visual capture alone cannot explain the increase in congruency of the evening sensation reports. Prior studies in short-term sensory learning have shown that repeated association of coincident stimuli can induce transient changes in both sensory cortical mapping and cortical activity levels. While these changes persist for a few hours after the presentation of the associated stimuli, they are fully reversible and disappear within 8–24 h (Rossini et al., 1994; Godde et al., 1996; Oliver et al., 2007). Thus, the within-day location changes we observed in early stages of this study may have arisen from reversible cortical changes which occurred because of the reinforcement of the visually captured sensory percepts as the participant interacted with various objects throughout the day. Given the sparse within-day trends for sensation quality, sensation quality may not be as influenced by visual capture or short-term cortical changes as sensation location.

Over the course of the 115-day study, the prevalence of within-day location changes decreased and long-term changes in location, intensity, naturalness, and quality emerged. The sensations became permanently aligned with the prosthesis sensors, such that the sensations matched the transduced information immediately upon system donning. The accumulation of experience with prosthesis-object interactions throughout the study enabled repeated multisensory associations of the evoked sensations with other sensory modalities and promoted passive learning of the sensation (Bogacz, 2007). In the interview, the participant also described active learning strategies, in which he purposefully practiced interpreting sensation. Over time, these passive and active learning mechanisms led to the long-term entrenching of the congruous sensory percepts.

The long-term somatosensory learning observed over the course of the study could have been driven by mechanisms related to cortical plasticity. The somatosensory cortex remains highly plastic through adulthood, and changes in sensory cortical representation following amputation are well-studied (Merzenich et al., 1984; Pons et al., 1991; Lund et al., 1994; Borsook et al., 1998; Huttenlocher, 2002; Weiss et al., 2004). Adult somatosensory plasticity is also a key driver of recovery after nerve injury (Bach-y-Rita, 1990; Lundborg, 2000; Fraser et al., 2002; Navarro et al., 2007; Knox et al., 2015), and, depending on the extent of the injury, the time courses associated with cortical reorganization can range from weeks (Merzenich et al., 1984; Borsook et al., 1998) to a year (Pons et al., 1991; Lund et al., 1994). In addition, prior studies have shown that both passive and active learning regimens in cochlear prostheses are associated with cortical plasticity (Tremblay et al., 1997, 1998; Fu and Galvin, 2007; Gentner and Margoliash, 2009; Sharma and Dorman, 2012). Analogous cortical plasticity mechanisms to those observed in cochlear prosthesis use may have driven the long-term ingraining of congruous artificial tactile percepts over the study duration. Future neuroimaging studies would be necessary to confirm whether the long-term perceptual changes shown here correspond to cortical changes indicative of plasticity.

As the participant became more familiar with the sensory feedback over the course of the study, higher-level sensory experiences began to emerge. The qualitative analysis demonstrates that the participant began interpreting sensory feedback from all sensors holistically as opposed to separately, providing the experience of stereognosis. Stereognosis is considered a complex, emergent property of the sensory experience that requires integration of tactile sensations, proprioception, and motor intent (Carlson and Brooks, 2009). Further, the ability to merge somatosensory information into a single object percept is dependent on familiarity with the object: people are better at identifying common objects like hammers than novel nonsense shapes (Klatzky et al., 1985; Ernst and Bülthoff, 2004). In addition, stereognosis of novel objects can improve upon training (Simmons and Locher, 1979). At early time points, the participant did not appear to experience stereognosis. However, in later interviews, the participant reported “global” percepts that matched the shape of objects he held most frequently, such as cups and bottles. Despite being familiar with these everyday objects prior to entry into the study, stereognosis only emerged over time after gaining repeated exposure to these object interactions and learning the sensory feedback. We hypothesize that the experience of stereognosis emerged as learning modified the participant’s sensory experience and the sensory feedback integrated with his prosthesis motor control.

We also observed an increase in the perceived naturalness of the sensation experience over the course of the study. Sensation naturalness is an important and controversial concept in somatosensory neuroprostheses. Typically, neuroprosthetics research groups discuss naturalness in the context of sensation quality. Electrical stimulation of the nerves typically results in unnatural feelings of paresthesia or “tingling” (Schady et al., 1983; Macefield et al., 1990; Kaczmarek et al., 1991; Geng et al., 2012; Perovic et al., 2013; Clark et al., 2014; Ortiz-Catalan et al., 2014; Tan et al., 2014; Saal and Bensmaia, 2015), although several stimulation approaches have been shown to improve the perceived naturalness of the artificial sensation quality (Tan et al., 2014; Valle et al., 2018). The participant rarely indicated the word “tingling” on the quantitative surveys throughout this study. Thus, the significant increases in sensation naturalness that we observed for two channels over time suggest that the concept of naturalness is related to familiarity with a particular sensation, regardless of its quality. In fact, while the participant occasionally described the naturalness of the sensation quality in the interviews, he more frequently described the naturalness of the sensation experience or of the prosthesis experience. He discussed how the more he used the prosthesis, the more natural the experience became. He also used the words “natural” and “normal” interchangeably, further corroborating the interaction of naturalness and familiarity or expectation. Based on this evidence, we believe that the naturalness of a sensation is a top-level interpretation of the normalness or familiarity of sensory information, and as such, may be impacted by other cognitive factors. Importantly, this increase in naturalness over time, which is likely related to passive learning of the artificial sensation, indicates that neuroprostheses do not have to perfectly reproduce sensory percepts to be useful. By simply approximating the correct inputs with electrical stimulation, cortical plasticity may assist in producing interpretable and natural sensations.

It is encouraging that the participant was able to undergo learning within the time course of this study. Unlike cochlear prostheses, whose users receive continuous auditory feedback from the time of implant, the participant in this study only received somatosensory feedback when he chose to wear the device. These choices and various interruptions throughout the study limited the continuity of his sensory feedback exposure. Because this is the first long-term home study of artificial somatosensory feedback, we do not know the extent or time course of washout due to intermittent periods of non-use. Despite these periods, we still observed significant changes in sensory perception. We hypothesize that the effects observed in this study would be further strengthened if there were fewer periods of non-use, but additional studies are needed to confirm or refute this hypothesis. Current studies in the neuroprosthetics field are attempting to understand the effects of manipulating stimulation parameters on sensation independently of any long-term learning effects (Clark et al., 2014; Graczyk et al., 2016, 2018a; Oddo et al., 2016; Wendelken et al., 2017; Valle et al., 2018). The results from this study indicate that learning strongly modulates sensory perception and should be controlled for in the laboratory setting. In addition, these changes in sensory feedback due to learning indicate that prosthesis users could benefit from training paradigms to promote active integration of sensory feedback into prosthesis utilization strategies.

We also do not know if there is a difference between periods of non-use in which the participant received no sensory feedback and periods of non-use in which the participant received erroneous or random sensory feedback due to malfunctioning system components. Our *post hoc* comparison of data from days in which the index sensor functioned at the beginning of the study to days in which it malfunctioned later in the study indicates that receiving incongruent or random feedback promotes maladaptive learning. This suggests that future studies should be careful to reduce or eliminate exposure to incongruent or erroneous sensory feedback in order to maximize useful sensory learning. Further, only limited information, pressure or aperture, was transduced from four sensors in this study. Additional work is necessary to quantify whether perceptual changes can extend to additional sensors, such as those required for a dexterous hand, or sensation types, such as textural features of an object.

### Implications of Psychosocial and Functional Changes Over Time

As artificial sensation was assimilated into the prosthetic hand experience and learned over time, the participant’s perceived functional ability and psychosocial outcomes improved, but with varying time courses. Several outcomes appeared to be predominantly influenced by the presence or absence of sensation instead of accumulation of sensation experience. For example, the participant performed better identification of foam blocks with sensory feedback than without at every testing interval. Similarly, specific subscales of psychosocial outcomes evaluated by the PEM, including self-efficacy, embodiment, social touch, and body image, reached a plateau in improvement within the first interval of usage. Given that our prior 2-week home study also showed improvement on these measures (Graczyk et al., 2018b), future studies of these types of specific psychosocial outcomes may not require extended home usage.

Embodiment also appeared to be more influenced by the presence of sensation than by time. Both the quantitative and qualitative analyses indicated that using the prosthesis with sensation led to increases in prosthesis embodiment. Embodiment is a complex phenomenon that includes both conscious and unconscious processes (de Vignemont, 2010b). The conscious perception of the body image (Gallagher and Cole, 1995; Gandevia and Phegan, 1999) was measured with several surveys, including the PEM and RHI, and demonstrated that the participant viewed the prosthesis as part of the body (Murray, 2004). The conscious perception of self-attribution, or ownership of the prosthesis as belonging to the self (Tsakiris and Haggard, 2005; Tsakiris, 2010), was also indicated by the participant’s usage of possessive pronouns to refer to the prosthesis as “my hand” in the interviews. Although we utilized primarily self-report surveys and interviews, which inherently measure conscious perception, our data also indicates that unconscious aspects of embodiment improved throughout the study. The reported changes in the perceived position of the phantom hand over time likely indicate a change to the body schema, which is the sensorimotor internal model of the body and an unconscious aspect of embodiment (Maravita and Iriki, 2004; Gallagher, 2005; Giummarra et al., 2007; de Vignemont, 2010a). The extension of the phantom toward the prosthetic fingertips could be considered a type of proprioceptive drift, a common indicator of embodiment in which the proprioceptive sense of the hand position moves to become aligned with the tool (Tsakiris and Haggard, 2005; Longo et al., 2008; de Vignemont, 2010b; Kalckert and Ehrsson, 2014). The alignment of the phantom with the prosthesis is evidence that the phantom successfully merged with the prosthesis (Giummarra et al., 2010).

Given that both tool embodiment and perceptual learning involve similar neural changes in sensory cortices (Iriki et al., 1996; Hoffman and Logothetis, 2009; Miller et al., 2017), the experience of embodiment may have been intricately linked to the sensory learning of the artificial somatosensory percepts. As the participant learned the artificial somatosensation, he may have refined his ability to integrate the artificial sensation into his existing body representation, leading to more efficient and accurate prosthesis actions. The participant’s reports of decreases in the attention required to use the prosthesis could indicate incorporation of the prosthesis into the body schema, since peri-personal space is prioritized in attention (Reed et al., 2006) and embodiment expands peri-personal space to include the peri-tool space (Galli et al., 2015; Miller et al., 2017). Interestingly, the decrease in phantom telescoping experience may have also been influenced by cortical changes that occurred through these learning mechanisms. Prior research has shown that telescoped limbs are associated with cortical remapping of distal limb segments onto nearby regions of the somatosensory cortex (Giummarra et al., 2007). The decrease in phantom telescoping could have occurred as sensory learning reorganized the sensory cortex to again distinguish the hand area from the arm area in the cortex.

In contrast to these fairly immediate effects of sensation, the holistic measures of the participant’s quality of life (OPUS QoL) and perceived disability (QuickDASH) improved but had not yet plateaued by the end of the 115-day study. Several measures of perceived prosthesis function, such as the PSFS and foam block confidence, similarly significantly increased through the study without reaching a plateau. This aligns with results from previous short-term training studies in which participants’ perceived function improved after two weeks of training with a sensory-substitution prosthetic system (Dietrich et al., 2012, 2018). Surprisingly, the participant’s in-lab performance of the foam block identification task with sensory feedback did not significantly improve over the course of the study, although it did trend upward. This may be due in part to the choice of functional task. Blindfolded recognition of foam blocks does not have a direct analog in the home setting and thus may not have been influenced by passive learning. The foam block test also does not have established validity or reliability, and it may not be sensitive enough to detect changes due to learning. Further, work in cochlear prostheses suggests that passive learning may take up to 12 months to occur (Watson, 1991; Fu and Galvin, 2007, 2012). If passive learning of somatosensory feedback occurs on a similar time course, it is possible that improvements in functional and psychosocial outcomes that are driven by sensory learning will not be detectable until later points in the learning process. Studies with longer-term follow-up are needed to confirm or refute this hypothesis.

The qualitative analysis demonstrated that the participant’s engagement with his prosthesis increased as he learned to utilize sensory feedback. He reported being more willing and more likely to use the prosthesis in performing skilled single-handed or bilateral tasks and in engaging in social interactions. He frequently described performing tasks with the sensory-enabled prosthesis that he did not do previously due to a lack of confidence in his ability to perform them correctly. For example, he became more willing to handle dishes with his prosthesis when unloading the dishwasher, because he believed that the sensation would give him information about slip and allow him to correct grasping errors before dropping a dish. However, quantitative indicators of prosthesis usage, such as the modified UEFS Task Completion metric, did not show significant increases over the course of the study. This may be because the specific tasks on the modified UEFS metric did not reflect the types of tasks the participant became more willing to do throughout the study. For example, the modified UEFS does not include any tasks related to social interactions (Jarl et al., 2014). Further, many tasks in the modified UEFS, such as buttoning a shirt or writing, require extensive mechanical dexterity of the hand and wrist. Performing these tasks with the single DOF hand provided to the participant could be difficult regardless of sensory feedback. In fact, the participant discussed that the mechanics of the sensory-enabled hand limited his ability to perform some tasks and mentioned a desire for a sensory-enabled dexterous hand.

If sensory feedback can increase the willingness to use a prosthesis or lead to more active use, this could have implications for the health and well-being of upper limb amputees. Approximately 28% of upper limb amputees reject their prosthesis, stating that they feel more functional without any prosthetic device than with one (Biddiss and Chau, 2007a, b). Sensory feedback could reduce the visual attention required to perform tasks with the device, which is a desired prosthesis improvement (Atkins et al., 1996). Thus, sensation may help reduce abandonment of prostheses by providing additional motivation to use a prosthetic device and improving perceived prosthesis function. In addition, relying solely on the intact contralateral limb often leads to overuse injuries in the contralateral limb and trunk (Burger and Vidmar, 2016; Postema et al., 2016). Active engagement of the prosthesis in activities through artificial sensory feedback could reduce overuse injuries, leading to enhanced quality of life and lower healthcare costs for this population.

## Conclusion

We studied the effects of 115 days of home-use of a sensory-enabled prosthesis on sensation experience, psychosocial outcomes, and prosthesis function in a single participant with acquired upper limb loss. Using mixed methods, we found that many aspects of the participant’s experience changed over the course of the study. Perception of sensation location and quality changed over time to better align with the multisensory information acquired through repeated prosthesis usage. These sensory changes likely resulted from active and passive learning mechanisms, indicating that cortical plasticity can mediate sensory learning even for artificial sensations produced by electrical stimulation. In addition, prosthesis embodiment, confidence, and other psychosocial measures improved significantly over the course of the study. These psychosocial impacts often appeared within a month of at-home usage, suggesting that sensory-prostheses can have rapid benefits for persons with upper limb loss. Finally, the perceived function of the prosthesis and active engagement of the prosthesis in tasks increased over the home trial. This study provides the first evidence that artificial somatosensation can undergo similar learning processes as intact sensation and highlights the importance of sensory restoration in prostheses.

## Data Availability

The raw data supporting the conclusions of this manuscript will be made available by the authors, without undue reservation, to any qualified researcher.

## Ethics Statement

All study devices and procedures were reviewed and approved by the U.S. Food and Drug Administration Investigational Device Exemption, the Cleveland Department of Veterans Affairs Medical Center Institutional Review Board, and the Department of the Navy Human Research Protection Program. All study procedures and experiments were performed in accordance with relevant guidelines and regulations of these institutions. The patients/participants provided their written informed consent to participate in this study.

## Author Contributions

IC designed the study, designed the analyses of quantitative sensory data, collected the data, performed the quantitative and qualitative analyses, interpreted the data, and wrote the manuscript. AG and LR designed the qualitative analysis, analyzed the qualitative data, and revised the manuscript. DT contributed to the study design and data interpretation, and revised the manuscript. EG designed the study, designed the analyses of quantitative and qualitative data, collected the data, performed the quantitative and qualitative analyses, interpreted the data, and wrote the manuscript.

## Conflict of Interest Statement

DT has patents on the electrodes (US Patent #6456866B1) and stimulation patterns (US Patent #9421366B2) related to sensory restoration. DT and EG also have a patent application on stimulation patterns related to sensory restoration (PCT/US2017/056070). The remaining authors declare that the research was conducted in the absence of any commercial or financial relationships that could be construed as a potential conflict of interest.
