# Supplementary material for: Learning of Artificial Sensation Through Long-Term Home Use of a Sensory-Enabled Prosthesis
Source: Front Neurosci. 2019 Aug 21;13:853. doi: 10.3389/fnins.2019.00853 (PMC6712074; doi:10.3389/fnins.2019.00853)
Supplement: Supplementary file 1 [file Table_1.DOCX]

Supplementary Material

**Supplementary Table 1: Timing, cause, and duration of study interruptions.**

| **Interval** | **Event** | **Cause** | **Duration (days)** |
| --- | --- | --- | --- |
| 1 | Interruption | Hardware breakage | 9 |
| 2 | Interruption | Illness | 3 |
| 2 | Interruption | Illness | 2 |
| 3 | Interruption | Skin irritation due to poor socket fit | 5 |
| 3 | Interruption | Illness | 11 |
| 3 | Interruption | Urgent personal matter | 1 |
| 3 | Interruption | Hardware breakage | 4 |
