# Supplementary material for: Learning of Artificial Sensation Through Long-Term Home Use of a Sensory-Enabled Prosthesis
Source: Front Neurosci. 2019 Aug 21;13:853. doi: 10.3389/fnins.2019.00853 (PMC6712074; doi:10.3389/fnins.2019.00853)
Supplement: Supplementary file 2 [file Table_2.DOCX]

Supplementary Material

Supplementary Table 2: Sensation quality descriptor words and their provided definitions. Italicized text is additional explanation of how the participant rated each descriptor.

| **Descriptor Word** | **Definition** |
| --- | --- |
| Intense | *Intensity is the strength or magnitude of the sensation. Participants were asked to rate the intensity on a scale from “no sensation” to “the most intense sensation imaginable.”* |
| Natural | A totally natural sensation would feel exactly like something you might experience in your intact hand. *Participants were asked to rate naturalness on a scale from “not natural (totally unnatural)” to “totally natural.”* |
| Unpleasant | Words used to describe very unpleasant sensations include “bothersome”, “miserable” and “intolerable.” Remember, sensation can have a low intensity but still feel extremely unpleasant, and some kinds of sensation can have a high intensity but be very tolerable. *Participants were asked to rate unpleasantness on a scale from “not unpleasant” to “the most unpleasant sensation imaginable.”* |
| Tingling | Words used to describe tingling sensations include “like pins and needles” and “prickling”. |
| Pressure | Words used to describe a pressure sensation include “heavy”, “weighted”, and “compression”. |
| Rough | Words used to describe a rough sensation include “like sandpaper” and “like Velcro”. |
| Electrical | Words used to describe electrical sensations include “shocks”, “lightning”, and “sparking”. |
| Contact touch | A phrase used to describe contact touch is “like resting my finger on a table”. |
| Sharp | Words used to describe sharp feelings include “like a knife”, “like a spike”, or “piercing”. |
| Cramping | Words used to describe cramping sensation include “squeezing” and “tight”. |
| Vibration | Please ignore the speed or pitch of the vibration when completing this scale. Words to describe vibration include “pulsing”, “buzzing”, “flutter”, “tapping”, or “like an electric toothbrush”. |
| Edged | “Edged” refers to the physical shape of the sensation, not sharpness. Phrases used to describe an edged sensation include “like the edge of a table” and “like pressing on the thin side of a butter knife”. |
| Movement | Words used to describe a movement sensation include “finger flexion” and “wrist rotation”, although the movement could be about any joint. *Participants were instructed that this descriptor was for sensations of finger or hand movements (i.e. proprioception).* |
