# Supplementary material for: Learning of Artificial Sensation Through Long-Term Home Use of a Sensory-Enabled Prosthesis
Source: Front Neurosci. 2019 Aug 21;13:853. doi: 10.3389/fnins.2019.00853 (PMC6712074; doi:10.3389/fnins.2019.00853)
Supplement: Supplementary file 3 [file Table_3.DOCX]

Supplementary Material

Supplementary Table 3: Full definitions for each theme and axial code generated through the qualitative analysis. Grey rows are the theme definitions. Italicized text provides further explanation of the coding structure.

| Theme | Node | Definition |
| --- | --- | --- |
| Sensation experience | Sensation experience | This theme encapsulates comments describing the experience of having sensation. This includes both what sensation feels like and how the participant’s opinions about sensation. |
|  | Sensation description | Comments about the location, intensity, type (modality/quality) or time course of sensation. Comments detailing specifics of the sensation or comparing the sensation from each of the sensors. Comments about the location, intensity, or type of sensation at a specific instance in time or at the time of the interview. |
|  | Stereognosis | Comments about merging or integration of sensations from multiple sensors into a single global or holistic percept. Comments about perceiving physical features of an object, such as size or shape, from sensation. |
|  | Preference for sensation | Comments demonstrating positive or negative views on the experiences with the sensation/ sensory prosthesis. Comments demonstrating that the subject “liked” or “preferred” sensation in certain conditions (or not). Comments about wanting to feel the sensation or activating the sensors solely for the purpose of feeling the sensation. |
| Learning | Learning | This theme includes comments about learning to interpret the sensory stimulation or use it to improve prosthesis control and task performance. |
|  | Usefulness of sensation | Comments about the ways in which the sensory feedback was (or was not) helpful for performing tasks with the prosthesis or comments about situations in which the sensation was useful (or not). Comments about the usefulness of sensation influencing his willingness (or lack of willingness) to use the prosthesis. Comments about the usefulness of the sensation in using the prosthesis, controlling the prosthesis, or performing certain functional or bilateral tasks (or not). Comments about the usefulness of sensation during social interactions. Comments about changes in the sensation due to pressing on sensors or activating the aperture sensor. Comments about how tasks changed (or did not change) the location, intensity, or type of sensation. Comments about how changes in pressure with object interactions changed the sensation experience. |
|  | Mechanisms of learning | Comments about acquiring new strategies for using the sensation or prosthesis. Comments about purposefully practicing or training in specific tasks or in differentiating sensations in order to improve abilities. Learning can be either passive or active. |
|  | Ease and attention | Comments about the ease of using the prosthesis or sensation and the speed of using the prosthesis or sensation. Comments comparing the ease or speed of use to past experiences. Comments about the ease or speed of using the prosthesis for carrying out tasks. Comments about the relative speed or ease of using the prosthesis compared to the intact hand. Comments about the focus or visual attention required to use the prosthesis or perform tasks with the prosthesis. Comments about how sensation changed (or did not change) the attention or focus required to use the prosthesis. Comments about the participant’s ability to use the prosthesis for tasks and interactions more efficiently, with less cognitive or physical effort, and/or less carefully. |
| Prosthesis engagement | Prosthesis engagement | This theme includes comments about the participant’s willingness to use the device, wear time of the prosthesis, use of the prosthesis for specific activities, or willingness to try activities with the prosthesis. It also includes comments describing the factors that influence his willingness to use the device. |
|  | Functional tasks | Comments about the ways in which sensation aided in prosthesis function (or not). Comments about the specific tasks or activities which could be performed with the prosthesis (or not). |
|  | Bilateral activities | *This node is a child of functional activities and a grandchild of prosthesis engagement.*  Comments about performing activities that require use of both hands (both the intact hand and the prosthesis). Comments about the willingness to do or try bilateral activities. |
|  | Interaction with others | Comments about using the prosthesis in social greetings and interactions, such as shaking hands or playing with children. Comments about the perceived ability to use the prosthesis in social settings or to interact with others (or not). |
|  | Confidence/ self-efficacy | Comments about the level of confidence the participant had with using the prosthesis or system components. Comments about the participant’s perception of their ability to successfully complete tasks. Comments about the types of tasks or interpersonal interactions or the conditions in which the participant felt confident (or lack thereof) with using the prosthesis. Comments about the confidence in using the prosthesis compared to the intact hand. |
| Embodiment | Embodiment | This theme includes comments related to limb ownership, body schema, body image, and perception of the phantom limb. |
|  | My hand | Comments in which the prosthesis is described as part of the body or belonging to the body, may contrast to a prosthesis that is only a tool. Comments showing ownership of the prosthesis (or not) or incorporation into the subject’s body representation (or not). Comments in which the subject uses possessive pronouns to refer to the prosthesis (or not). |
|  | Naturalness | Comments about the perceived naturalness of using the system, sensation, or prosthesis. Comments stating that use of the system, sensation, or prosthesis was similar to the experience of using the intact hand (or not). Comments about the intuitive use of the device (or not). Comments about his expectations for sensation and the prosthesis and how his experience matches his expectations (or not). |
|  | Perception of phantom limb | Comments about the phantom position or posture. Comments about phantom limb telescoping. Comments about the relationship between sensation or sensory feedback and the phantom limb. Comments about the perception of or the experience of the phantom limb. |
| System operation | System operation | This theme includes comments about the technical aspects of wearing the sensory-enabled system. |
|  | Problems | Comments about technical difficulties with the system or breakages of system components. Comments about interference of the sensation or system components with usage of the prosthesis or control of the prosthesis. |
|  | Ease of use of system | Comments about the ease or speed of using and setting up the system. Comments about the speed or frequency of calibration of the sensory stimulation settings. |
