# Supplementary material for: Learning of Artificial Sensation Through Long-Term Home Use of a Sensory-Enabled Prosthesis
Source: Front Neurosci. 2019 Aug 21;13:853. doi: 10.3389/fnins.2019.00853 (PMC6712074; doi:10.3389/fnins.2019.00853)
Supplement: Supplementary file 4 [file Data_Sheet_1.PDF]

Supplementary Material

Supplement 1: Semi-structured interview questions

A brief semi-structured interview was conducted during each laboratory visit to determine subject experiences with the system during the preceding month. Each interview lasted between 20-40 min in duration. These interviews were videotaped and transcribed for subsequent qualitative analysis. The interviewer asked the following direct questions, and follow-up questions or clarifications were used to probe the participant's responses:

1. What did you think about the sensory feedback this month?
2. How would you describe your experiences this week?
3. Was the sensory feedback useful?
4. Did the sensation change the way you viewed or thought about your prosthesis?
5. Did the sensation change the way you used the prosthesis?
6. What did the sensation feel like?
7. Did the sensations change throughout the day?
8. Did the sensations change throughout the month?
9. Did you have any problems with the system?
10. Did you prefer having the sensory feedback?
11. **For interviews following intervals 2 and 3:** How did the sensory feedback this month compare to previous month(s)?
