# Supplementary material for: Learning of Artificial Sensation Through Long-Term Home Use of a Sensory-Enabled Prosthesis
Source: Front Neurosci. 2019 Aug 21;13:853. doi: 10.3389/fnins.2019.00853 (PMC6712074; doi:10.3389/fnins.2019.00853)
Supplement: Supplementary file 5 [file Data_Sheet_2.PDF]

Supplementary Material

Supplement 2: Comparison of sensory changes when prosthesis sensors worked correctly and malfunctioned

Several sensors malfunctioned or may have malfunctioned over the course of the study (Supplementary Figure 1). The thumb sensor worked correctly throughout the study. The middle finger sensor also worked throughout the study, except on days 36 and 37. These sensors' analyses are completely presented in the main text.

The index finger sensor (channel 2) failed early in the study. On day 36, after 13 days of system usage, the logged sensor data demonstrated highly aberrant sensor activity for channel 2, likely due to a problem with the wiring of the sensor cable. The participant's daily diaries also confirmed the sensor breakage, as he reported that sensation became sporadic or erratic from this day onward. However, the participant continued reporting sensation location, quality, and intensity for this channel through day 51 (Supplementary Figure 1A). On day 52, the sensor catastrophically failed such that no sensory feedback was provided for the remainder of the study. Statistical comparisons were made for the perceived sensation location and quality when the sensor correctly transduced information versus when it malfunctioned. The alignment of the perceived location to the prosthesis sensor location when the sensor was working versus not were compared using tests of 2-proportions. The sensation VAS descriptors when the sensor was working versus not were compared using two-sample t-tests.

There were significant differences in reported sensation location for channel 2 when the sensor was working versus non-working (Supplementary Figure 1B). When the sensor was working, the sensation was significantly more likely to be aligned with the prosthesis sensor location than not (test of 2 proportions, $p < 0.001$). In contrast, the sensation location was more likely to be misaligned with the sensor location than aligned when the sensor was malfunctioning, although this difference was not significant (test of 2 proportions, $p = 0.184$). There were no significant differences in sensation descriptor words when the index sensor was working versus non-working (2-sample t-tests, p = 0.051, 0.654, 0.493, 0.506, 0.147, and 0.708 for “intense”, “natural”, “pressure”, “contact touch”, “vibrating”, and “movement”, respectively) (Supplementary Figure 1C).

The aperture sensor (channel 4) may have failed towards the end of the study, but this could not be confirmed due to discrepancy between the sensor logs and participant reports. For days 1-58, the aperture sensor was confirmed to work both from the logged sensor data and the participant's diary reports. However, the sensor logs demonstrate little to no variance in sensor value after day 58. This suggested that the sensor never triggered changes in the sensory feedback as the participant moved his prosthesis. Due to the low resolution of the sensor log vs neurostimulator during analog-to-digital conversion, it is possible that the neurostimulator was able to detect differences in the sensor value that the sensor log could not. Because of this, it is possible that the participant perceived useful changes in sensory stimulation as he moved his prosthesis during daily tasks. Further, in contrast to the participant's diary accounts of index sensor breakage, the participant never reported in his daily diary that the aperture sensor malfunctioned or stopped providing sensation. After the possible aperture sensor breakage was identified, we asked the participant whether the sensor malfunctioned

at any time during the study, and he replied that it did not. Thus, it is probable, but impossible to confirm, that the aperture sensor was functioning throughout the entire study. To cautiously account for the uncertainty, only data from days 1-58 for channel 4 were presented in the main text.

In this Supplement and Supplementary Figure 1, we present an additional analysis of channel 4. We assume that the aperture sensor did not malfunction during the study, and combine the datasets from days 1-115 to analyze trends in sensation quality for channel 4. In this analysis, within-day changes are evaluated using paired t-tests, and across-day changes are evaluated using linear regression. For the within-day comparisons, there were no significant differences in sensation descriptor words between the mornings and evenings for the aperture sensor (paired t-tests, $p = 0.558$, 0.248, 0.237, 0.128, 0.057, and 0.360 for “intense”, “natural”, “pressure”, “contact touch”, “vibrating”, and “movement”, respectively) (Supplementary Figure 1D). Over the full 115-day study, the participant reported significant increases in the intensity and naturalness of sensation (linear regression, $p = 0.001$ and $p < 0.001$, respectively) (Supplementary Figure 1E), as he had for the two working tactile channels (channels 1 and 3, see main text). Surprisingly, his rating of the proprioceptive descriptor, “movement”, which trended upward, did not significantly increase over the course of the study (linear regression, $p = 0.162$). The participant’s ratings of tactile descriptors did not trend together: his rating of “pressure” increased although not significantly (linear regression, $p = 0.072$), his rating of “contact touch” significantly increased (linear regression, $p = 0.006$), and his rating of vibration significantly decreased (linear regression, $p = 0.006$).
