# Supplementary material for: Learning of Artificial Sensation Through Long-Term Home Use of a Sensory-Enabled Prosthesis
Source: Front Neurosci. 2019 Aug 21;13:853. doi: 10.3389/fnins.2019.00853 (PMC6712074; doi:10.3389/fnins.2019.00853)
Supplement: Supplementary file 6 [file Data_Sheet_3.pdf]

Supplementary Material

Supplement 3: System Usage Theme Node Report

Throughout the study, the participant experienced technical difficulties with and breakages of the system. These problems included: failure of the neurostimulator to turn on, breakage of the index finger pressure sensor, and failure of connectors (connection between prosthesis sensors and control unit). Most of these issues were single instance problems, and were resolved before continuing the trial. However, he experienced a few problems consistently.

One problem he noted was difficulty in calibrating the pressure sensors:

> *"According to how hard you squeeze on the block [points at calibration block] or how sensitive they decide to be, I still have to watch some. Because, if I squeeze to where I can get sensation, sometimes it's squishing the sandwich like that [fully closes prosthesis]."* [T3]

He also noted that the cabling would interfere with his stimulation calibration routine:

> *"When I'm setting it up, calibrating, going through, might feel 1 and it feels a little weak, might not feel 2, then I move it [gestures at magnetic connector]. A lot of times I think it was [channel] 4 that I'd have trouble with feeling, that I'd have to move back and forth. And when I'd get it just right where I could feel [channel] 4, then the others would be a little bit stronger than what they were."* [T2]

In general, however, he stated that he didn't need to spend much time calibrating the stimulation, because the sensations were consistent:

> *"It's even gotten to the point that umm I put it on in the morning then, I go through my calibrate and don't really have to adjust anything there. Staying pretty, pretty set."* [T1]

> *"But now I know about where to set it. I've wore it long enough. I knew where to set it. And it's to the point now that I just get up in the mornings and turn the box on, run through the calibration, and don't even have to adjust things. So very seldom that I've had to adjust it."* [T3]

Another problem he discussed was accidently turning off the system:

> *"And there was days the off button would get hit, so then I'm opening up the bag and having to check [mimes that action]. I think that more happened, umm, getting in the vehicle, putting the seat belt on. Umm, going to the bathroom [chuckles], messing with the bag and everything [gestures at where fanny pack sits on right hip], going to the bathroom and all. It was more apt to get tapped and hit and shut off and then [grasps intact hand with prosthesis] a little bit later I'd notice no sensation, so let's check here first [mimes opening and looking into the fanny pack] and make sure it wasn't that."* [T3]

"There's a couple times where I was wearing it that the box got shut off by accident and everything, just like sensation would get shut off because [gestures at where fanny pack sits on right hip] of where it's at, the fanny pack and all. Somebody left-handed [gestures at where fanny pack would sit on the left hip for a left-arm prosthesis] may not have as many problems, because like seat belt- they're not going to be hitting it [mimes putting on seatbelt]. I'm hitting it, doing different things trying to put my seatbelt on." [T3]

However, the main problem he discusses is variability in the intensity of the sensation. He talks about the sensation "spiking" on occasion, forcing him to recalibrate the box. He ascribed this problem to weakness in the wiring of the system. The wiring by the participant's percutaneous leads includes a magnetic breakaway to prevent pulling of the leads if the wiring catches on anything:

"I think it's more right there [grabs cabling at shoulder and bends it back and forth]. Cuz I feel something now when I move it around. It, you know, will bounce around a little bit." [T2]

"Just the one I normally have, trying to [rocks magnetic connector back and forth]. Cuz that has that slide and it's come, you know, it's come unplugged a couple times, but having to move that back and forth sometimes to get sensation in all of them." [T2]

"Every now and then, the magnetic connector and I'd have to push it back and forth some and everything [mimes this action] to get them all working at a even level. Because, it's got just enough move that if you move it one way, well, these two are strong, but that one's weak; you move it the other way, well those two are strong but that one's a little weak. So, it was just a matter of trying to [makes magnetic connector adjusting motion]." [T3]

He noted that the spikes were more surprising than painful:

"Umm, when it'd jolt real high it wasn't too nice [laughs] and everything. But it wasn't anything that hurt. It was just more a-a-a shock, I wasn't expecting it to jump like that and everything. And then, like I said, I think most of that was because of the wiring [points at cables on socket]." [T3]

Often, this spiking behavior would cause interference with his myoelectric control:

"And sometimes it was a matter of fine-tuning them so that they didn't close automatically, because I do contract sometimes with the sensation." [T3]

"There'd be days it'd get stronger throughout the day and everything and I might have it wanting to close on me [closes and opens prosthesis] and everything, but it'd be towards the end of the day. And I don't necessarily know whether it was sensation actually getting stronger or from wearing it all day- maybe the arm and the muscles, getting a little bit fatigued and wanting to close [closes and opens prosthesis] when I felt the sensation. And maybe feel stronger [shrugs]. I mean that, I have no idea on." [T3]

He stated that this problems reduces his confidence in using the device to perform tasks or interact with others:

"There was a few days I wrote down that it was spiking and a few days I was having troubles cuz my other arm was acting up and it would close and I'd be having trouble opening it. So,

you know, those are days no I didn't feel comfortable grabbing anything fragile, shaking somebody's hand, or anything like that cuz I didn't know whether it was going to open or not or try to squeeze close tighter." [T2]

However, when asked whether "all these problems with the hand and the sensors and the wiring... impact your view of your preference for the sensation?" he stated:

> *"Not too bad. There was days it would be frustrating when, you know, the sensors decide you got to squeeze real hard to feel something. Or the magnet would move [points at magnetic breakaway], and it might jump a little bit. Or you might lose sensation in one or another and everything. But, still would rather have it than not have it."* [T3]
