# Supplementary material for: Learning of Artificial Sensation Through Long-Term Home Use of a Sensory-Enabled Prosthesis
Source: Front Neurosci. 2019 Aug 21;13:853. doi: 10.3389/fnins.2019.00853 (PMC6712074; doi:10.3389/fnins.2019.00853)
Supplement: Supplementary file 7 [file Image_1.pdf]

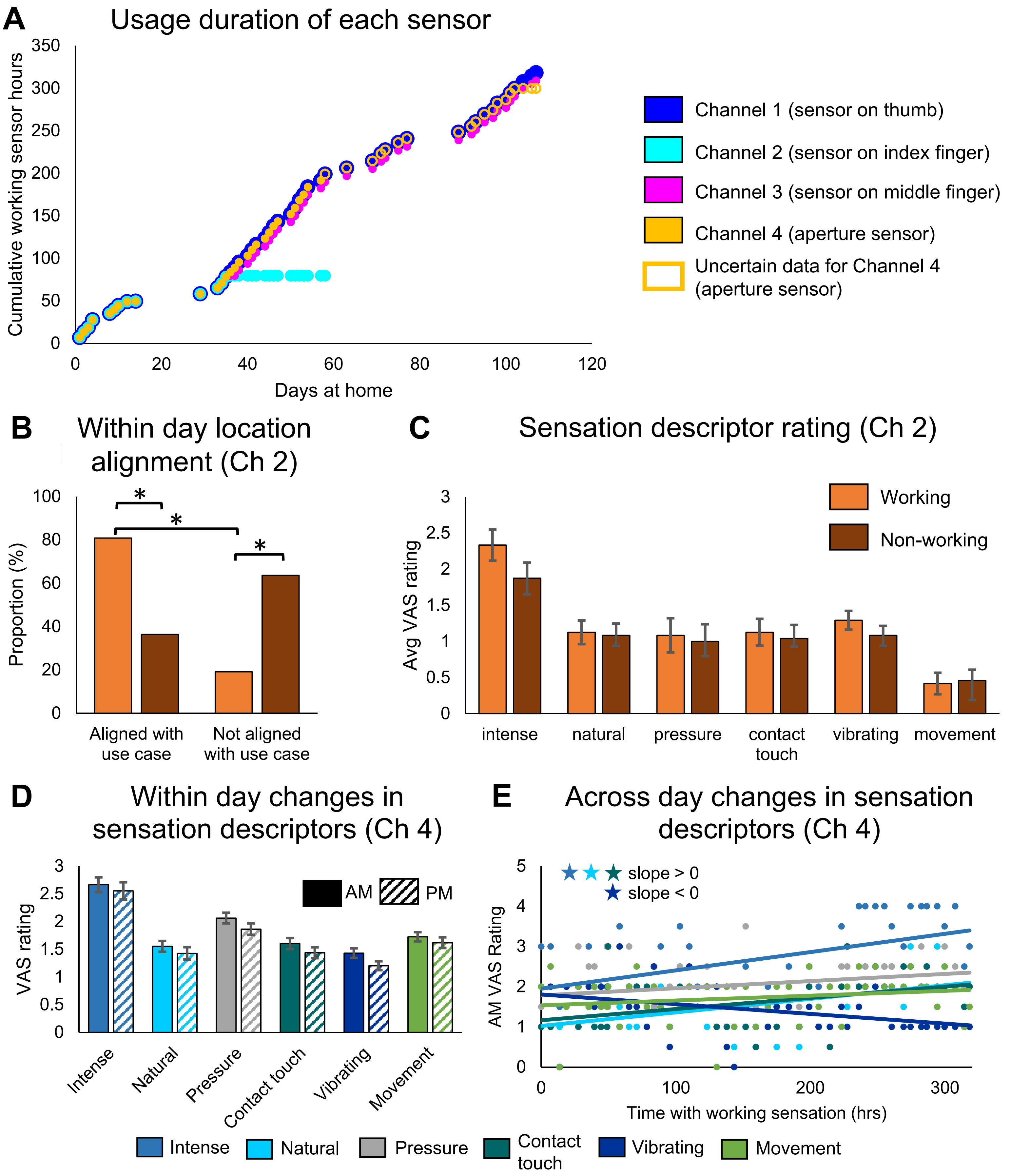

Supplementary Figure 1: The effect of working vs non-working prosthesis sensors on artificial sensory percepts. **(A)** Sensor function varied over the course of the study for each of the sensors (n = 115 days in total). **(B)** Within-day location alignment with the prosthesis sensor for channel 2 (index sensor) when the sensor was working (orange, n=12 days) vs non-working (red, n = 11). Asterisks denote significant differences (p<0.05). **(C)** Sensation quality for channel 2 when the sensor was working (n=12 days) vs non-working (n = 11 days). **(D)** Within-day changes in sensation quality for channel 4 (aperture sensor) across the duration of the study (n = 48 days). **(E)** Across-day changes in sensation quality for channel 4 across the entire duration of the study (n = 48 days). Stars denote significant changes over time (p<0.05).
