# Supplementary material for: Learning of Artificial Sensation Through Long-Term Home Use of a Sensory-Enabled Prosthesis
Source: Front Neurosci. 2019 Aug 21;13:853. doi: 10.3389/fnins.2019.00853 (PMC6712074; doi:10.3389/fnins.2019.00853)
Supplement: Supplementary file 8 [file Image_2.pdf]

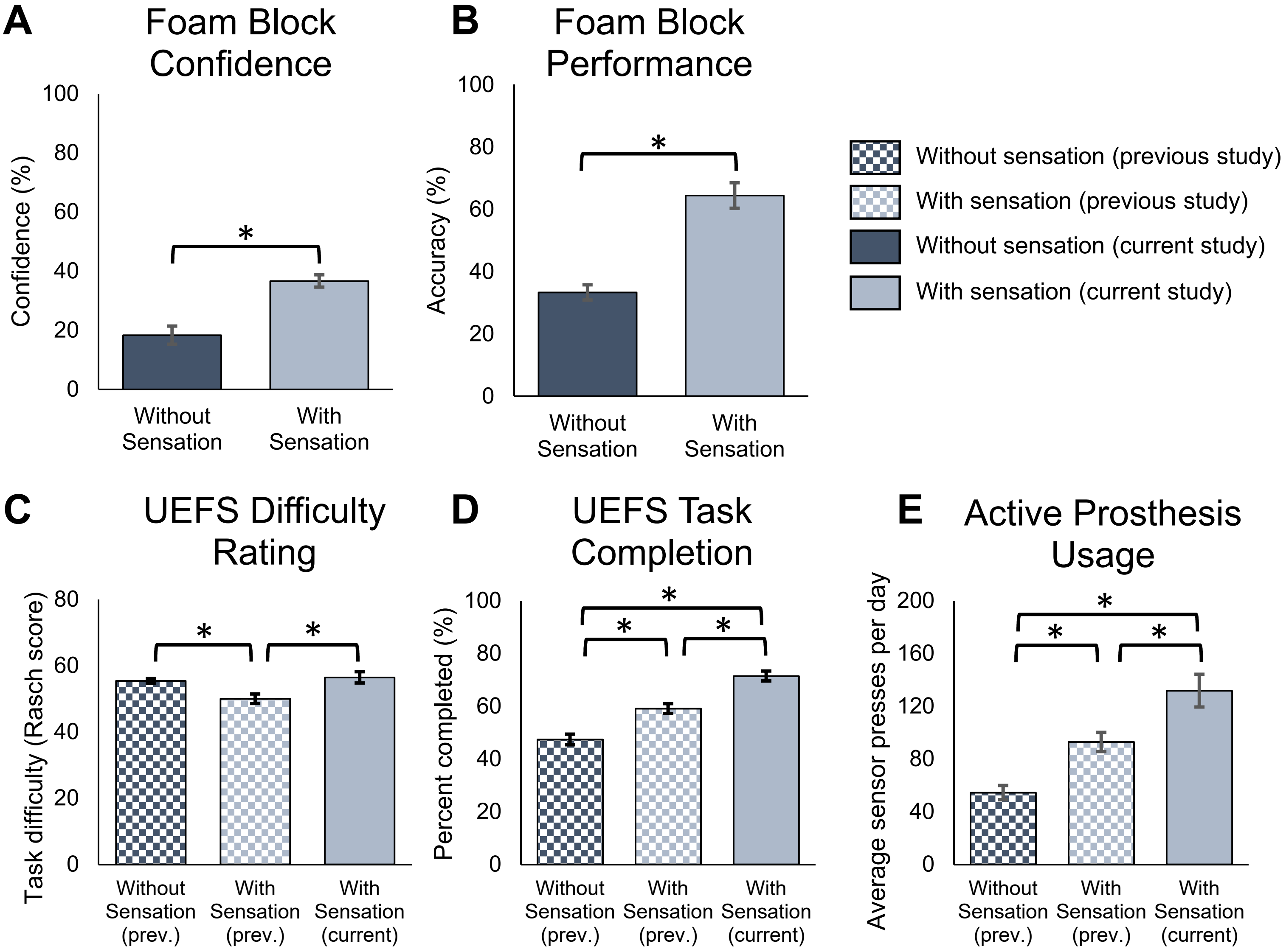

Supplementary Figure 2: Comparison of functional outcomes with and without sensory feedback. **(A, B)** Foam block outcomes were pooled across time points (data points include in-lab testing after Intervals 1, 2, and 3) and compared between the sensation "on" and "off" conditions (n = 4). **(A)** The participant's confidence in performing the task was significantly higher with sensation enabled (paired t-test, $p < 0.001$). **(B)** The participant's ability to perform the task was significantly higher with sensation enabled (paired t-test, $p = 0.003$). **(C, D)** UEFS outcomes were pooled within this study and compared to UEFS outcomes from Stages 1 and 3 (sensation disabled) and from Stage 2 (sensation enabled) from a previous study reported in (Graczyk et al., 2018b) (n = 24, 13, 9 for without sensation previous, with sensation previous, and with sensation current, respectively). **(C)** The participant's perception of task difficulty was significantly worse with sensation in the previous study, but ratings did not differ between this study and without sensation in the previous study (1-way ANOVA followed by Tukey, $p = 0.001$). **(D)** The participant completed significantly more tasks with sensation in the current study than either with or without sensation in the previous study (1-way ANOVA followed by Tukey, $p < 0.001$). **(E)** Average sensor presses per day were pooled for all days within this study and compared to average sensor presses from Stages 1 and 3 (sensation disabled) and from Stage 2 (sensation enabled) from a previous study reported in (Graczyk et al., 2018b). The participant actively used his prosthesis significantly more when he had sensory feedback in the current study than either with or without sensation in the previous study (1-way ANOVA followed by Tukey, $p < 0.001$) (n = 24, 13, 48 for without sensation previous, with sensation previous, and with sensation current, respectively).
